# Dataset on wastewater quality monitoring with adsorption and reflectance spectrometry in the UV-vis range

**DOI:** 10.1038/s41597-025-05459-x

**Published:** 2025-07-25

**Authors:** Pierre Lechevallier, Günter Gruber, Vojtěch Bareš, Nicolas Neuenhofer, Laura Waldner, Abhinit Mahajan, Lena Mutzner, Jörg Rieckermann

**Affiliations:** 1 Department of Urban Water Management, Swiss Federal Institute of Aquatic Science & Technology (Eawag), Dübendorf, 8600 Switzerland; 2 Institute of Urban Water Management and Landscape Water Engineering, Graz University of Technology, Graz, 8010 Austria; 3 Department of Hydraulics and Hydrology, Czech Technical University in Prague, Prague, 166 29 Czech Republic

**Keywords:** Environmental monitoring, Hydrology

## Abstract

A major challenge in wastewater and sewer system monitoring is the development of advanced sensing technologies to improve standard pollutant measurement and allow real-time online detection of emerging contaminants. This study presents a dataset from a 25-week measurement campaign comparing a novel hyperspectral imaging system to state-of-the-art ultraviolet-visible (UV-vis) sensors. The dataset includes 5801 hyperspectral images of raw wastewater, measurements of temperature, ammonium, flow, turbidity, pH, and UV-vis absorbance spectra, as well as 533 grab samples analyzed for conventional pollutants. We also gathered 86 samples after four rain events and analyzed them for twenty organic chemicals, providing insights into the impact of wet weather on pollutant levels. The data collection and processing methodologies are detailed, along with visualizations and analysis. Despite difficulties in the maintenance of some sensors, in particular the ion-selective electrode for ammonium measurement, the dataset’s high temporal resolution, the time span of 25 weeks, and the extensive range of analyzed pollutants make it a valuable resource for advancing the field of urban water management.

## Background & Summary

Monitoring urban wastewater flows and pollution levels is required to ensure optimal mitigation strategies. While robust solutions exist to monitor wastewater levels, velocities, and flows in urban drainage systems (UDS), it is still challenging to measure concentrations of pollutants with a high temporal resolution^1,2^. Among available technologies for *in-situ* wastewater pollution monitoring, sensors based on absorbance spectrophotometry have been widely used in the last 20 years^3,4^. Utilizing variations of the water’s spectral signature, they can estimate within a matter of seconds a wide range of pollution variables, such as chemical oxygen demand, nitrate, nitrite, or suspended solids^5^. However, due to their contact with wastewater, they require regular maintenance, making their implementation very costly and often even impossible on a larger scale^6^. In addition, environmental conditions and sensor-specific properties can also compromise data quality^7^. Finally, spectrophotometers have a small measurement window of a few square millimeters, making them prone to sampling bias. Therefore, considering the challenging conditions of urban drainage systems, we must develop innovative sensors.

Recently, non-contact spectrophotometric techniques based on light reflection measurement have gained attention as a potential improvement to absorbance spectrophotometers. Contrary to the measurement of light absorption, the measurement of light reflection does not require any contact with the wastewater, which greatly reduces sensor fouling and decreases maintenance costs. Russell *et al*.^8^ and Agustsson *et al*.^9^ investigated this approach using spectrophotometers to capture light reflection. In a previous study based on laboratory measurements, we improved their approach by investigating the use of a hyperspectral imaging system instead of a spectrophotometer^10^. We were able to estimate different pollution variables with comparable precision to ultraviolet-visible (UV-vis) spectrophotometry. Specifically, we generated mixtures of real and synthetic wastewaters and used a data-driven model to estimate turbidity and the concentration of soluble compounds such as organic carbon, nitrogen, and phosphate. We found that turbidity, ammonium, and total dissolved nitrogen were especially promising, as they were estimated with precision below 10%. However, since our experiments used synthetic wastewater mixtures in a controlled lab environment, like Agustsson *et al*.^9^, there are several limitations that need to be addressed before this approach can be applied in real-world conditions.

Firstly, one cannot directly transfer the results from the lab setup, which has a defined geometry and a calm water surface, to the monitoring of real wastewater under turbulent flow conditions in UDS. Second, the number of ground truth samples was rather low, which prohibits the use of advanced data-driven modeling methods. With the exponential growth in computing power, new machine learning methods are being constantly developed, making faster and more precise calibration of data-driven models possible^11^. While these sophisticated methods enable the solving of complex and non-linear problems, they require ever-increasing amounts of data^12^. To make reflection-based methods more accurate, we need to gather large, reusable datasets of hyperspectral images of wastewater that include qualitative and quantitative reference data and metadata.

In this publication, we present a unique dataset that we collected over a 25-week monitoring campaign aimed at comparing the performance of state-of-the-art absorption-based spectrophotometers with newer reflection-based techniques for wastewater monitoring. To address the limitations of laboratory data representativeness, data quantity, and data quality, we conducted this study using raw wastewater from a nearby sewage trunk continuously brought through a flume to replicate flow conditions in sewers. We installed five water quality sensors along the flume to monitor the wastewater’s turbidity, ammonium, pH, and UV-vis absorbance at a temporal resolution of two minutes. In addition, a hyperspectral imaging system acquired visible-near infrared (VNIR) images of the wastewater surface with a resolution of 30 minutes. During the 25 weeks of the experiment, 533 reference samples were manually collected and analyzed in the laboratory. In summary, this dataset provides the potential to assess the performances of reflectance- and absorption-based techniques and train data-driven models with 2-minute-resolution sensor data as well as qualitative laboratory reference measurements.

## Methods for data collection

**Fig. 1:**
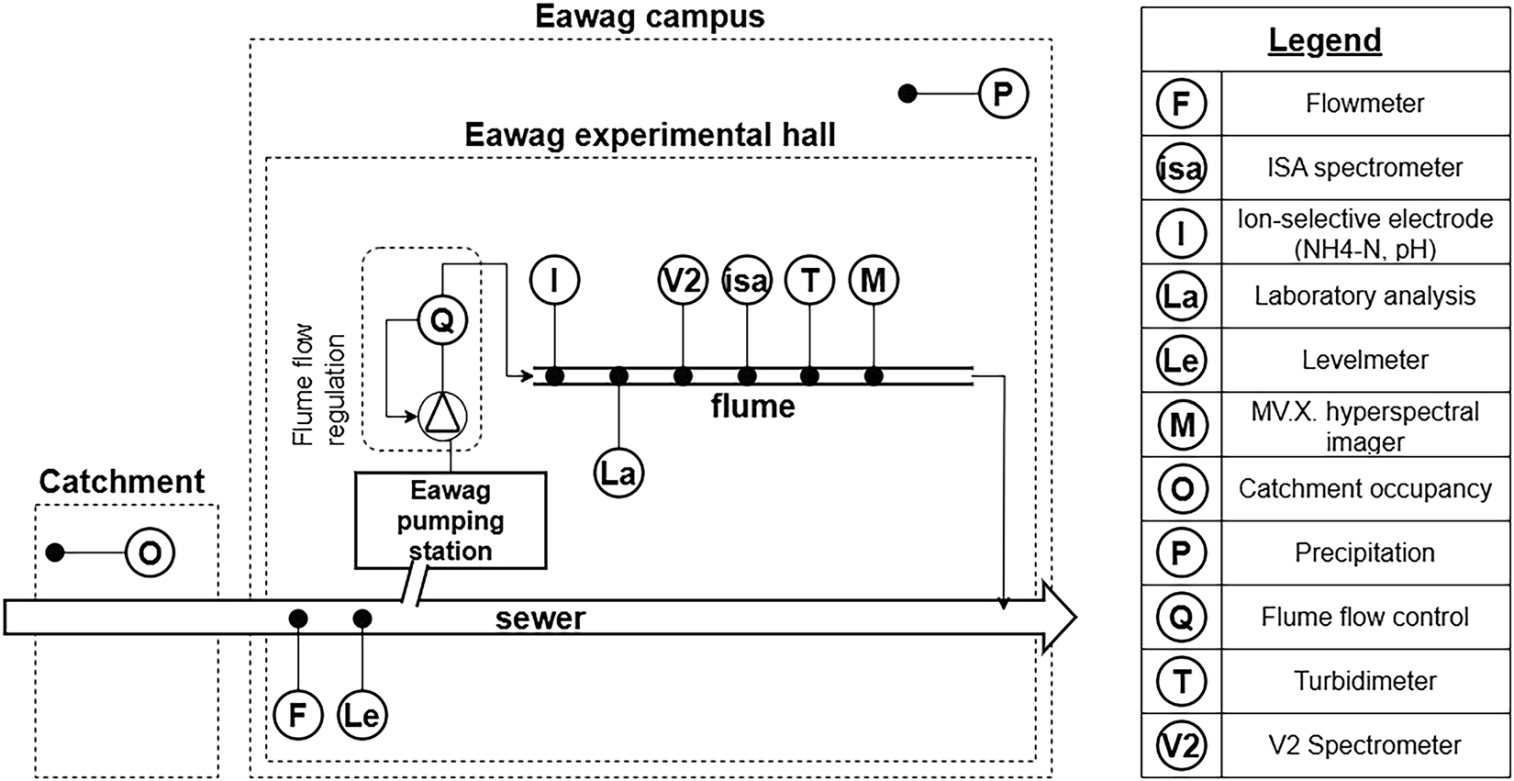
Overview of the experimental design and sensor location of the flume experiment.

We designed this experiment to track pollution variations in raw urban wastewater over several months. To that end, we directed wastewater from a nearby sewer through a flume. The flume had state-of-the-art sensors monitoring the wastewater’s turbidity, the ammonium concentration, and the wastewater absorbance in the UV-vis range. In addition, we installed an innovative sensor capturing VNIR hyperspectral datacubes (i.e., 3D images) of the wastewater surface. We also collected data on precipitation, population density within the catchment, and the amount of wastewater in the sewer system. Figure 1 provides an overview of each data source location.

### Catchment characteristics and wastewater origin

The wastewater studied in this experiment comes from a medium-sized catchment of 71.2 ha, which totals 24,782 population equivalents (PE). As shown in Supplementary Figure 1, the catchment is drained by a combined sewer system and a separate sewer system. The catchment comprises the cities of Dietlikon (7,845 PE, 2022), Wangen-Brütisellen (8,000 PE, 2022), parts of Dübendorf (5,903 PE, n.a.), and parts of Wallisellen (3,034 PE, 2021) (E. Shilyaeva, personal communication, 6.10.2023; L. Barth, personal communication, 18.10.2023). The catchment area features diverse land uses, including residential areas, stores and warehouses, garages, streets, and industries (e.g., IKEA, Coca-Cola, Luzi AG (fragrances), Esslinger AG (construction), and Röhm AG (plastics)). Agricultural fields, allotment gardens, forests, a vineyard, and the airport of Dübendorf (only used for military and leisure air traffic) can be found in the surroundings of the catchment.

For the flume experiment, we pumped raw wastewater from the nearby trunk sewer. The sewer is a 1,000-mm-diameter round concrete pipe with a 1.5% slope. In this pipe, the dry weather flow displays a distinctive pattern between workdays and weekends or holidays, as shown in Supplementary Figure 2. Typically, the minimum flow of 60 L/s occurs at night between 01:00 and 03:00, and it reaches its maximum flow of 110 L/s around 07:00. A second flow maximum of about 100 L/s occurs around 21:00.

### The flume setup: an open channel for continuous monitoring of raw urban wastewater

To monitor wastewater pollution with various sensors, particularly the reflection-based imaging system, we brought wastewater through a flume, a 4 m long and 10 cm wide rectangular channel with a 2.5% longitudinal slope. We installed a submersible pump (Flygt NS 3085.160 SH-253-400V-2.4 kW, Xylem, France) in a pit to pump wastewater from sewers to the flume. This pit received wastewater from the adjacent sewer (see Supplementary Figure 1). We used a flowmeter (Promag 53 W, Endress + Hauser, Switzerland) to regulate the pumping rate, maintaining a constant flow rate of 12 m^3^/h and an approximate surface velocity of 1 m/s in the flume. Under those conditions, the flume’s water depth remained constant at around 10 centimeters, which was required to ensure stable measurement conditions for the imaging systems. Pictures and technical drawings of the flume are available in Supplementary Figures 3–7.

To reproduce sewer light conditions while minimizing interference from external light in spectroscopic measurements, the flume was covered with an optical black nylon tissue coated in polyurethane (Thorlabs, Germany). Additionally, we fixed black PET sheets to the flume walls in the area where the hyperspectral imaging system monitored the wastewater surface to minimize light reflection from the hyperspectral illumination on the flume walls. Hanging black wooden barriers also provided optical insulation between those areas and the rest of the flume.

The experiments lasted 172 days (8/05/2023-26/10/2023), during which the flume operated with minimal interruptions. We shut it off once or twice per week for maintenance and sensor cleaning, which took thirty to sixty minutes, and seven times to address unexpected issues like pump clogging. Supplementary Figure 8 presents an overview of stops for flume maintenance and disruptions.

### Sensors installed in the flume

#### Hyperspectral imaging system

**Fig. 2:**
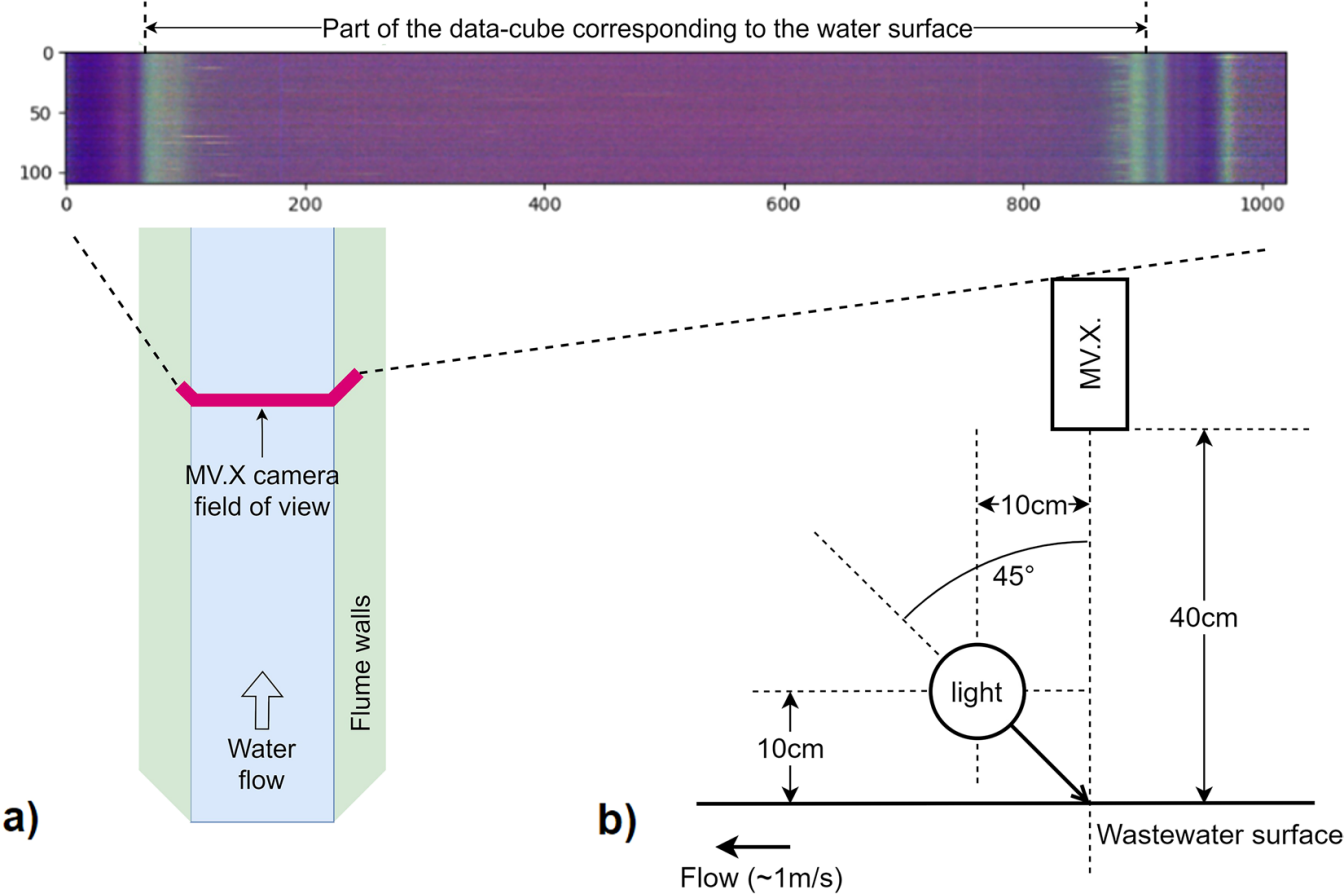
(**a**) MV.X imager’s field of view in the flume, and visualization of the typical raw hyperspectral datacube. (**b**) Schematic representation of the hyperspectral acquisition camera and light position relative to the wastewater surface.

We used the MV.X hyperspectral imaging system (Headwall Photonics, USA) to obtain hyperspectral images of the wastewater surface. The MV.X device is water-resistant (rated IP66 and IP67), making it suitable for use in a flume. It functions as a push-broom camera, employing a line scan technique to measure hyperspectral datacubes. It captures data with a spectral resolution of 2 nm spanning from 400 nm to 1000 nm, corresponding to the visible and near-infrared range. We oriented the camera’s field of view perpendicular to the water flow, covering the entire width of the wastewater surface and a small portion of the flume walls (see Fig. 2a). The spatial resolution was about 0.1 mm. We acquired each measurement during 10 s with an exposure time of 100 ms and an analog gain of 13.9, resulting in a datacube of 1,020 pixels, 100 lines, and 300 spectral bands. Due to the size of each datacube (about 65 MB), we restricted the measurement frequency to one hour until July 25, 2023, at 09:00 and 30 minutes afterwards, but we could potentially measure at a higher frequency with sufficient storage capacity.

We used an LED source (EFFI-FLEX-HIS-X2-100-910-970-TR-P1-LS-CYL-ELS-24V, Effilux, Germany) with continuous spectrum wide-band (400–800 nm) light to illuminate the wastewater surface. This illumination system is designed specifically for push-broom hyperspectral imagers. It generates a highly focused light line that covers the visible and near-infrared bands. It was positioned at a 45° angle and a 10 cm height relative to the wastewater surface to maximize diffuse light intensity reflected towards the camera while minimizing direct reflections. Figure 2b details the position of the MV.X imaging system and illumination relative to the wastewater surface.

#### UV-vis spectrophotometers

**Fig. 3:**
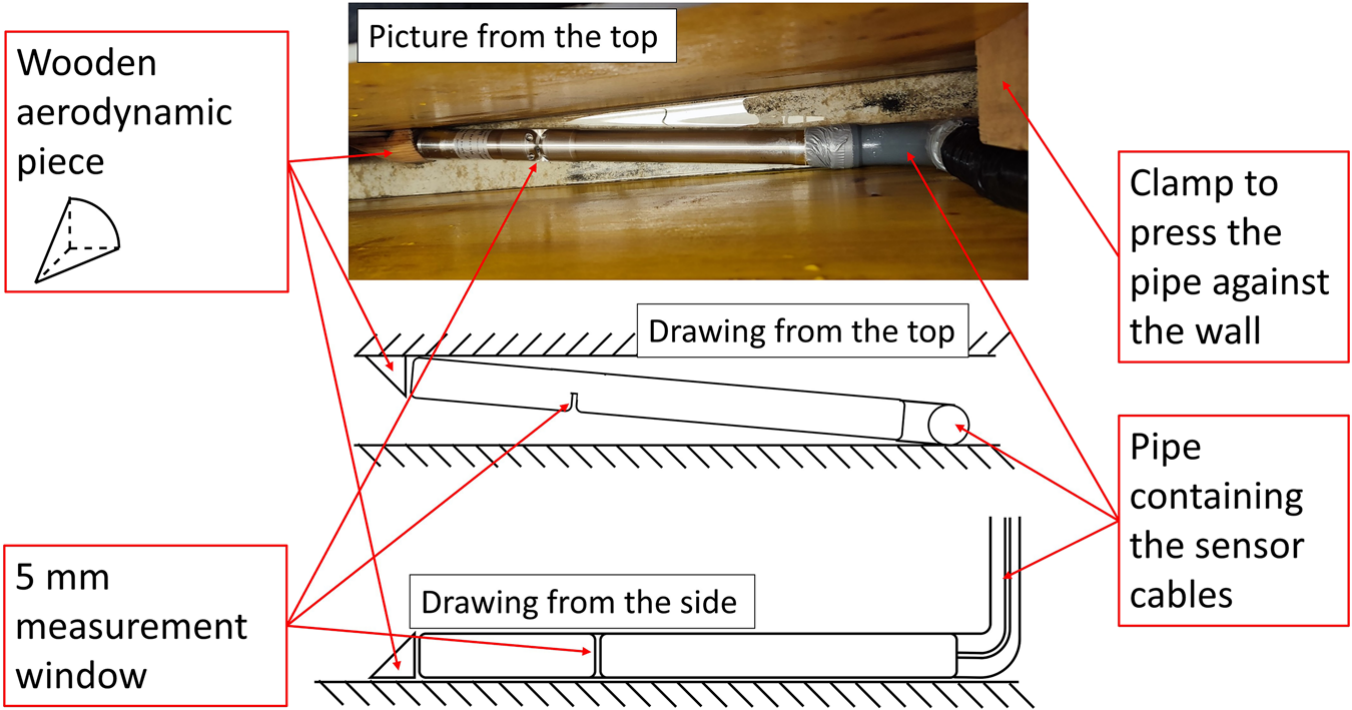
Description of the Spectrolyser installation. Note: in the picture, the sensor measurement window is parallel to the bottom, which is not the configuration used for the flume experiment. Instead, the sensor was rotated so that the measurement window is perpendicular to the bottom, as depicted in the sketches.

To compare the performance of hyperspectral imaging with that of state-of-the-art spectrometric technologies, we installed two UV-vis spectrophotometers in the flume. First, a V2 Spectrolyser (s::can, Austria) with an optical window of 5 mm was installed at the bottom of the flume (Fig. 3). The device is built from stainless steel and is equipped with a xenon flash lamp. It measures wastewater absorbance in the range between 200 and 735 nm, with a spectral resolution of 2.5 nm. Throughout the entire experiment, we measured wastewater absorbance every 2 minutes.

We took different measures to ensure that the accumulation of solids transported by the wastewater (sand, toilet paper, etc.) did not disturb the measurements. To prevent clogging at the front of the sensor, we first mounted a wooden piece with a hydrodynamic cylindrical shape against the left wall of the flume. Secondly, we added a bent cylindrical housing at the back of the sensor to contain the sensor cables that were otherwise accumulating toilet paper, ensuring a smooth flow of wastewater against it. This cylindrical element was maintained against the right wall of the flume with clamps. Third, we installed the sensor with the measurement window oriented perpendicular to the flume bottom.

Additionally, we used the Spectrolyser’s integrated pressurized air cleaning system every fifth measurement, i.e., six times per hour, to prevent clogging of the optical window and biofilm growth. Pressurized air cleaning was programmed to last 10 seconds and to finish 10 seconds before the next measurement to allow enough time for any remaining air bubbles to disperse. Finally, once or twice a week, the sensor was cleaned by hand with a water jet, scrub, and ethanol. System checks with distilled water performed before and after the experiment verified that the probe remained functional and no drifts in absorbance were found.

This dataset contains not only raw absorbance spectra measured by the probe but also estimations of four pollution indicators: biological oxygen demand (BOD), chemical oxygen demand (COD), total suspended solids (TSS), and nitrates (NO_3_)). We used the global calibration INFLUBODV150, delivered by the manufacturer and suited for raw wastewaters. We also measured the wastewater absorbance with a second sensor, the ISA spectrophotometer (Go-Systemelektronik, Germany). The sensor, also made of stainless steel, measures absorbance in the 200 to 706 nm range with a 2 nm resolution. This sensor, unlike the Spectrolyser, has an adjustable optical path length. We calibrated the probe with local wastewaters and found the optimal width to be 4 mm, which ensures the measurement of absorbance within the sensor’s linear range. We installed, cleaned, calibrated, and operated the sensor in the same manner as the Spectrolyser. Details about the ISA installation and management are available in Supplementary Figure 9 and Supplementary Table 1.

#### Ion-selective electrode and pH meter

We installed the ISEmax ion-selective electrode (CAS40D, Endress + Hauser, Switzerland) in the flume’s inlet chamber because it was too large to fit in the 10 cm wide flume channel (Supplementary Figure 7). This inlet chamber is a 50-cm-wide cylinder used to break the turbulence of the water arriving from the top before it enters the flume. The sensor head was equipped with electrodes for the measurement of ammonium nitrogen (NH_4_-N) and pH and a temperature sensor. Similar to the Spectrolyser and ISA, we cleaned the sensor manually once or twice a week with a water jet and scrub. However, data validation revealed only a limited correlation with reference laboratory measurements of ammonium, as detailed in the section “Technical validation”. Therefore, the data from the ISEmax sensor should be used with caution.

#### Turbidimeter

**Fig. 4:**
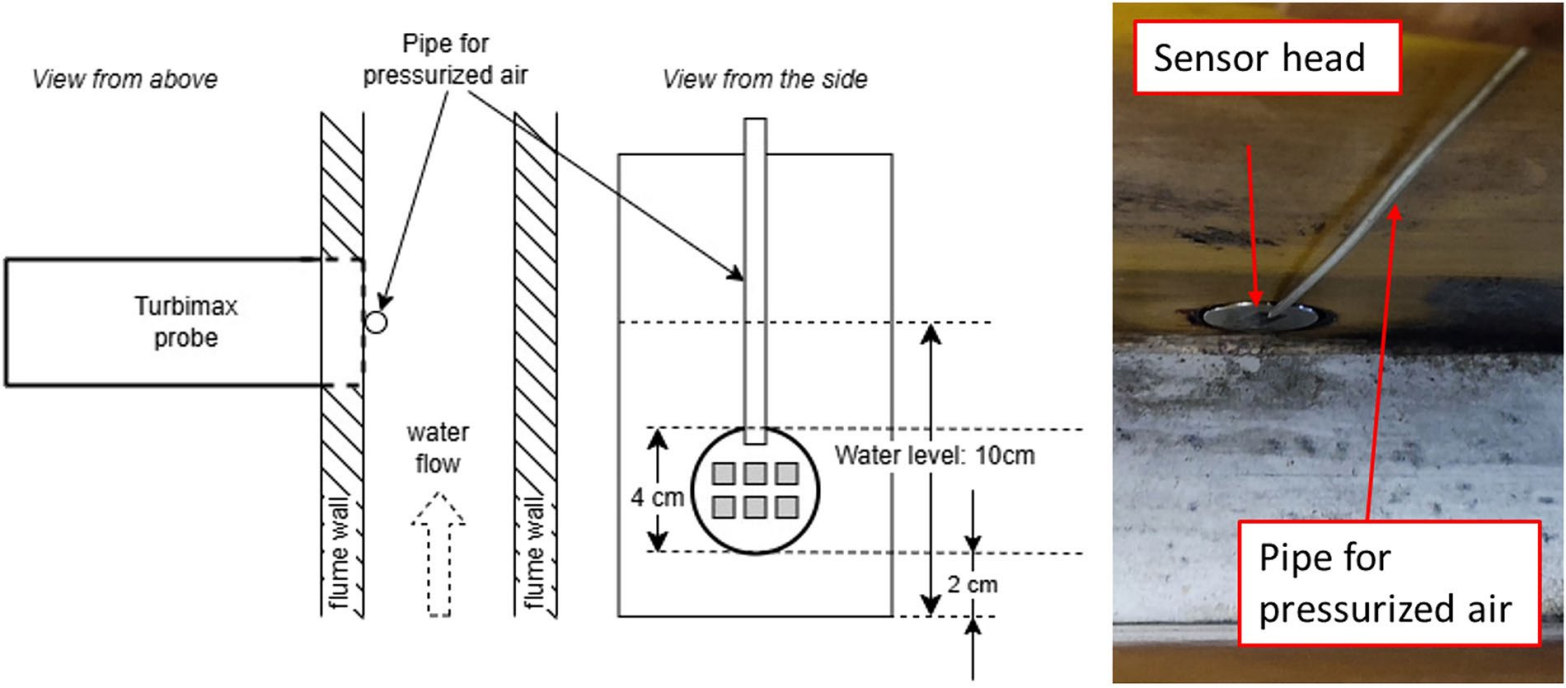
(left and middle) Schematic representation of the Turbimax installation in the flume wall. (right) Picture of the sensor from the inside of the flume.

We mounted the Turbimax probe from Endress + Hauser (CUS541D, Switzerland) to align the sensor’s head, which contains the measurement windows, precisely with the flume wall (Fig. 4). The sensor measures turbidity every two minutes. We performed sensor cleaning twice a week using a water jet, scrubs, and ethanol. Additionally, we added a pressurized air cleaning after 12/06/2023 at 9:00 to prevent sensor drifts. Data before this timestamp shows drifts. We set this cleaning to occur every 10 minutes for 10 seconds, effectively eliminating all drift. Between 05/07 and 10/07, the pressurized cleaning was not activated, and drifts were measured once again.

### Wastewater sampling for reference laboratory measurements

#### Sampling plan

To capture the daily, weekly, and seasonal variability of wastewater pollution, as well as the influence of dry and wet weather conditions, we grabbed wastewater samples regularly across the experimental campaign. We collected the samples at either the exact hour (xx:00) or the half-hour mark (xx:30) to synchronize with the MV.X hyperspectral imager. In total, 533 samples were analyzed. We collected the majority of samples (n = 513) between 6:00 and 20:00, sampled three full nights (20:00–6:00) with a two-hour resolution using the autosampler (see (M3) below), and collected five more samples between 20:00 and 24:00.

Because the wastewater used in this study originates from a combined sewer system, stormwater inflow is expected to substantially influence the reflectance and absorbance measurements. To estimate the extent of stormwater contribution, we decomposed the flow data into dry and wet components using STL decomposition^13^. Rainfall-runoff was observed on 25% of the monitoring days (see Supplementary File 1 for details). During these wet weather periods, we collected 199 samples for laboratory analysis, representing 37% of all reference samples.

Five pollution variables were measured in the laboratory for all samples: turbidity, phosphates (PO_4_-P), NH_4_-N, total dissolved nitrogen (TDN), sulfates (SO_4_-S), and dissolved organic carbon (DOC). We also measured TSS, total organic carbon (TOC), and total nitrogen (TN) for 45 of those samples. For 86 wet weather samples plus 3 field blanks (collected with nanopure water in the last three rain events), we analyzed 20 organic chemicals with liquid chromatography coupled with high-resolution tandem mass spectrometry (LC-HRMS/MS). More information about the sampling location, temporal distribution over the duration of the experimental campaign, and sampling time is available in Supplementary Figures 10–12.

#### Sampling methodology

The sampling methodology (M) varied depending on the sample analysis required.

- (M1). For all samples where TSS and/or organic chemicals were not measured, samples were collected manually at the beginning of the flume with a 50-mL syringe connected to a Ø10 mm plastic pipe. We divided the 50 mL directly into two vials. 35 mL were used to measure the turbidity, and the other 15 mL were filtered with Chromafil GF/PET 0.45 µm (Macherey-Nagel, Switzerland) that had been rinsed before use to look for soluble compounds.
- (M2) We collected the samples for organic chemical analysis during four rain events using a cooled MAXX TP5C autosampler (Mess-u. Probenahmetechnik GmbH, Germany) filled with 24 glass bottles. The autosampler grabbed samples at the same location as the manual grab samples described above. We manually triggered automatic sampling when the sewer flow increased above its dry weather baseline, approximately 1 hour after the start of rain, and stopped it either when the flow returned to its baseline level or when we filled all glass bottles. Each sample was 250 mL, taken either every 10 minutes as a single sample (first two rain events, 21.06.23 and 14.10.23) or as five time-weighted composite samples of 50 mL each, taken every 2 minutes (last two rain events, 20.10.23 and 24.10.23). At the end of each rain event, we transferred the samples into i) two half-filled 20-mL glass vials for freezing at −20°C and subsequent organic chemical measurement; ii) a 35-mL vial for turbidity measurement; and iii) a 15-mL vial after 0.45 m filtration for soluble compounds analysis.
- (M3) The MAXX autosampler sampled two nights (07-08/09/2023, 08-09/09/2023, and 27-28/09/2023) at a two-hour resolution. We handled those samples similarly to M1.
- (M4) We collected the samples that required TSS analysis at the end of the flume, where it was more convenient to grab two liters at once. We prepared 50 mL as M1 from these two liters and poured 1 L into a plastic bottle for homogenization and subsequent TSS, TOC, and TN analysis.

#### Measurement of traditional pollution indicators: turbidity, TSS, DOC, TOC, TN, TDN, NH4-N, PO4-P, and SO4-S

In the 30 minutes following collection, the samples were pre-processed and either further analyzed (for turbidity and TSS) or stored at 5°C for later analysis (remaining pollution indicators). Table 1 summarizes the pre-processing and measurement methods for each indicator. For the analysis requiring a filtration, we used Chromafil GF/PET 0.45 µm filters (Macherey-Nagel, Switzerland). We diluted the samples, when necessary, to be within the range of the measurement method. To measure TSS, TOC, and TN, we homogenized 1 L of the samples with an Ultra-Turrax (IKA, Germany) (15.000 rotations per minute for 2 minutes). To measure TSS, we used 0.4 m glass fiber filters (Macherey-Nagel, Switzerland).

**Table 1:** Information about each pollution variable measurement: sample pre-processing, measurement method, and accuracy.

Water quality variable	Sample pre-processing	Measurement method	Instrument	Accuracy
DOC	filtration dilution if necessary	TOC Analyzer (DIN EN ISO 20236, 2023)	Shimadzu TOC-L CSH	0.5 mg/L
NH_4_-N	filtration dilution if necessary	Flow Injection Analysis (standard method 4500-NH3, EPA 600/4-79-020, 1983)	Flex/Lachat QC8500	0.2 mg/L
PO_4_-P, SO_4_-S	filtration dilution if necessary	Ion-chromatography (DIN EN ISO 10304-1, 2009)	Metrohm 930 Compact IC Flex	< 0.1 mg/L
TDN	filtration dilution if necessary	TOC Analyzer (DIN EN ISO 20236, 2023)	Shimadzu TOC-L CSH	0.4 mg/L
TN	homogenization dilution if necessary	TOC Analyzer (DIN EN ISO 20236, 2023)	Shimadzu TOC-L CSH	0.4 mg/L
TOC	homogenization dilution if necessary	TOC Analyzer (DIN EN ISO 20236, 2023)	Shimadzu TOC-L CSH	0.5 mg/L
TSS	homogenization	Filtration (200 mL) DIN EN ISO 11923:1997	Ø 90 mm glass fiber filter 0.4 µm	5%
Turbidity	None	Turbidimeter (DIN EN ISO 7027-1, 2016)	Hach TL2300	2%

#### Measurement of organic chemicals

We selected a set of twenty organic chemicals (see Table 2) based on four criteria. First, we chose substances with high prevalence, i.e., high occurrence and concentration in UDS. Second, we selected organic chemicals coming from different urban sources. Third, we opted for organic chemicals for which we anticipated different source-specific dynamics in the catchment. Fourth, we selected polar substances with Log(K_ow_) ≤5 to ensure dissolution and transport in the water phase. Supplementary Table 4 presents details about typical uses and Log(K_ow_) of the selected organic chemicals.

**Table 2:** Summary of the twenty organic chemicals analyzed.

Municipal (+industrial) wastewater	Stormwater	Both
Acesulfame	1,3-diphenylguanidine	4-&5-methylbenzotriazole
Caffeine	6PPD-quinone	Benzotriazole
Cyclamate	Hexa(methoxymethyl) melamine (HMMM)	N-N-diethyl-3-methylbenzamide (DEET)
Candesartan	2,4-D	
Citalopram	Diuron	
Diclofenac	Carbendazim	
Hydrochlorothiazide	2-methyl-4-chloro-phenoxyacetic acid (MCPA)	
Triclosan	Mecoprop-p 2-n-octyl-4-isothiazolin-3-on (OIT)	

Additional information, including use and Log(K_ow_) are presented in Supplementary Table 4.

To analyze these organic chemicals, we left the frozen wastewater samples to thaw at room temperature. We then pipetted 1.2 mL of each sample into a 1.5 mL high-performance liquid chromatography (HPLC) vial, taking care to avoid settled particles. We centrifuged these vials for 10 minutes at 5,000 rpm and 10°C. Finally, 800 μL of the supernatant was pipetted into another HPLC vial.

For organic chemical quantification, we added structure-identical isotopic-labeled standards (ISTDs) to each sample to achieve a final concentration of 400 ng/L. ISTDs were available for all analytes, except for caffeine, cyclamate, citalopram, triclosan, 1,3-diphenylguanidine, HMMM, OIT, and 4- and 5-methylbenzotriazole. For analytes without an ISTD, we used the ISTD of the analyte with the most similar retention time for quantification. For standard addition, we spiked varying concentrations of the analyte standard (500 ng/L and 2,500 ng/L) into aliquots of one sample collected during low flow and one sample collected during high flow. This allowed us to calculate relative recoveries and eliminate matrix effects. For the calibration curve, we added varying amounts of these reference standards to Evian water (mock matrix), covering a range of 0.5–7,500 ng/L (first measurement series) and 0.5–10,000 ng/L (second measurement series). Anticipating that the concentrations of acesulfame, caffeine, and cyclamate would exceed the calibration range, we diluted aliquots of all samples 100-fold before adding the ISTDs and reference standards.

We identified target analytes using LC-HRMS/MS in two measurement series. In the first series, we measured samples taken on 21.06.23 and 24.10.23, and in the second series, we measured samples taken on 14.10.23 and 24.10.23, as well as dilutions. In the second series, the sensitivity of the instrument was lower, though still in an acceptable range. We directly injected the samples into a 6495 C triple quadrupole LC/MS system (Agilent Technologies) for quantification, using electrospray ionization in both positive and negative modes. For chromatographic separation, an Acquity UPLC HSS T3 column (3 × 100 mm, 1.8 μm particle size) and an Acquity UPLC HSS T3 VanGuard precolumn (2.1 × 5 mm, 1.8 μm particle size) (Waters Corporation) were used. We formed the LC gradient by varying the mixing ratio of acidic nanopure water and acidic methanol, both solvents containing 0.1% formic acid. We initiated it with 100% nanopure water for 1 minute, followed by a 17.5-minute linear gradient to 95% MeOH. We then ran the system with 95% MeOH for 3.5 minutes before reestablishing the initial conditions in 0.5 minutes (Supplementary Table 5). Supplementary File 2 contains additional information on the chemical analysis, including the settings (Supplementary Table 6) and the acquisition method (Supplementary Table 7) of the mass spectrometer.

For quality control of the measurements, we measured the Pharma-Mix17 (NEOCHEMA GmbH) at the beginning and end of the measurement series. To detect potential carry-over, we measured blanks (nanopure water) at the beginning and end, blinds (Evian water + ISTDs) at every eight samples, and calibration standards (500 ng/L or 750 ng/L) between samples of each rain event.

### Other data sources: sewer water level, rainfall, electrical conductivity, and population data

#### Sewer level and flow

We used a level meter and area-velocity flow meter to measure the amount of wastewater in the sewer. The sensors were installed 1 meter before the inlet of the pumping pit, from which wastewater was provided to the flume. An ultrasonic i3 sensor (Nivus, Germany) measured the level, while a CS2 Correlation Wedge Sensor (Nivus, Germany) measured the flow velocity. The flow and level measurements are unreliable between 27/08/23 at 00:45 and 18/09/2023 at 9:00 because a large stone blocked the velocity sensor.

#### Precipitation

We obtained rainfall data from the Swiss National Air Pollution Monitoring Network station (NABEL, BAFU and Empa) located on the Eawag campus (47.404841, 8.608490, Dübendorf, Switzerland). They used the Pluvio2S (OTT, France) precipitation sensor to obtain precipitation quantities in mm (resolution: 0.001 mm) every 10 seconds. This sensor utilizes a weighing principle, collecting both liquid and solid precipitation in a container with a 200 cm^2^ opening. The change in weight is measured with a highly precise, long-term stable load cell. Temperature changes on the weighing mechanism were compensated by an integrated temperature sensor. A filter algorithm is implemented to prevent distortion of measurement results, such as those caused by wind influences. The precipitation intensity is then calculated from the difference between two successive measurements. These values are finally accumulated to derive the total precipitation.

#### Electrical conductivity

Electrical conductivity was not monitored in the flume, but it was measured for the same wastewater in the primary clarifier of the Eawag wastewater treatment plant. The sensor, an E53 Electrodeless Conductivity Analyzer (GLI International, U.S.A.), was not managed by our teams, and no information is available about the sensor maintenance and data validation. As for the ISEmax sensor, the absence of regular pressurized air cleaning probably led to the accumulation of biofilm on the sensor head. We included the data from this sensor in this publication because the relative variations of the sensor measurement can still be useful for analyzing single rain events.

#### Occupancy: High-resolution catchment population

The study used mobility data from KidoDynamics^14^, a company that specializes in telecommunication data transformation. Such data have already been used to analyze the spread of COVID-19^15,16^. They use a method of filtering, cleansing, and anonymizing data to analyze mobility patterns and produce precise population data in a specific area. The data complies with the General Data Protection Regulation due to the implementation of various anonymization procedures, including Secure Hash Algorithm 1 anonymization, K-anonymity, and statistical noise application. For this study, we retrieved hourly occupancy data, i.e., population density, from the catchment area. The calculation procedure does not provide occupancy data for the hours of 23:00 and 00:00. Those missing data points can be extrapolated, as night occupancy is not anticipated to fluctuate significantly. Occupancy data are owned by the company and can be made available on request.

#### Sensor logbook and other information

We kept a logbook for each sensor used in this study. For every special event involving a sensor, we reported the start and end timestamps of the event, whether the data points are considered valid or not, and a brief explanation of what happened. Furthermore, in a dedicated logbook, we reported interruptions in the flume’s operations.

Finally, for each day of the experiment, we specified in a dedicated table whether the day was a holiday, a weekday, or a weekend, as well as whether it was during the summer holidays.

## Methods for data pre-processing: compilation, unification, and automatic data validation

This dataset contains data from different sources, including sensors, laboratory measurements, and external sources. We compiled the data into CSV files, keeping the raw hyperspectral datacubes in their original ENVI format. In this compilation process, we unified timestamps, we used a structured naming convention, and we performed automated datapoint validations. In this section, we also provide a brief explanation of the ISA data preprocessing and of the extraction of a spectral signature from the raw hyperspectral datacubes. Finally, we detail the specific pre-processing used for the organic chemicals data.

### Timestamp unification

Due to the drifting of some sensors’ clocks, it was not possible to ensure perfect synchronization. During the experiment, we synchronized the clocks of the flume sensor twice a week. When preprocessing the data, we rounded all timestamps to the nearest minute. This approach was considered acceptable for two reasons. First, wastewater quantities and qualities, except for intense rain events, do not vary a lot from minute to minute. For example, average absolute flow variations are less than 1% per minute, while average turbidity variations are 1.4% per minute. Second, since sensors were not all positioned at the exact same location in the flume, they were not exposed to the exact same wastewater at a specific point in time, making it practically impossible to ensure perfect comparability of the sensor measurements.

### Structured naming conventions

We used a structured naming convention to guarantee the uniqueness of all file and column names. The general convention is the following:

- File naming convention: sensor-location_sensor-name_variable-name
- Column naming convention: sensor-name_variable-name_unit

Supplementary Table 2 provides a table that summarizes all the possibilities for data origin, sensor name, variable name, and unit. For laboratory measurements, we used “lab” for all sensor names instead of the data origin. Finally, we kept the original names of the raw hyperspectral datacubes.

### Automated sensor data validation

All sensor data generated in this study include at least one column containing information about the datapoint validity, except for the hyperspectral datacubes that were all considered valid. Laboratory data were all considered valid. Depending on the data type and available knowledge, between one and three validation steps were performed:

1. Verification of flume operation: For all sensors installed in the flume, timestamps where the flume was stopped (gathered in the flume logbook) were flagged as invalid data points.
2. Verification of sensor operation: For all sensors for which we kept a logbook, timestamps where the sensor was not working were flagged as invalid data points.
3. Verification that data are within the expected range: For sensor data, a range delimiting the minimal and maximal acceptable measurements was defined to remove outliers. This range was determined through visual inspection of the raw data and knowledge of the raw wastewater quantities and daily variations. Supplementary Table 3 includes a table displaying the applied validity range.

### Preprocessing of ISA data

**Fig. 5:**
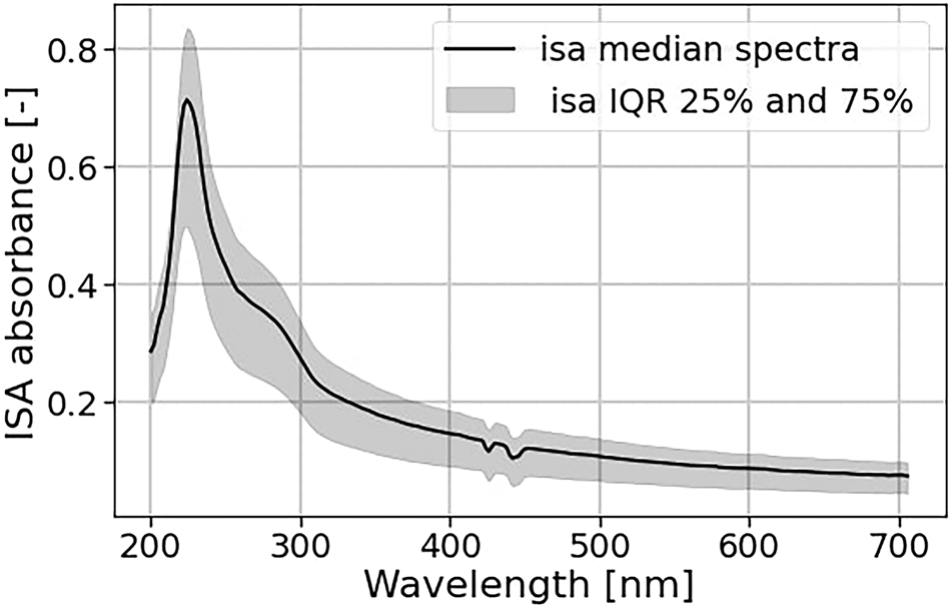
Median value of the raw ISA absorbance spectra.

ISA data requires an extra preprocessing step because the absorbance values in the low UV region must be discarded. According to the sensor supplier, the wavelengths prior to the absorption maximum can be considered as invalid due to the high absorption of the optical fiber in the UV range. For this dataset, the maximum occurs between 224 and 230 nm, as shown in Fig. 5. Therefore, we considered the range 230–706 nm as valid.

### Preprocessing of MV.X hyperspectral datacube for spectral signature extraction

This dataset includes the raw hyperspectral datacubes and a spectral signature extracted from each. This signature is a reflectance spectrum representative of the wastewater surface. There are different ways of performing this extraction, and we present here a simple procedure. It follows a series of four preprocessing steps inspired by common hyperspectral datacube processing^17,18^.

First, we convert the datacubes to reflectance by normalizing them with a dark and white reference. We acquired the white reference using a Zenith polymer diffuse reflectance standard, which has a 99% reflectance between 350 nm and 1,500 nm (SphereOptics, Germany). We positioned the reflectance standard at the water’s height in the flume. Second, we reframe the datacubes to retain only the area and the wavelengths of interest. The area of interest corresponds to the wastewater surface. The wavelengths of interest range between 400 and 800 nm, corresponding to the wavelengths of the illumination system. Third, we employ a pixel selection method to eliminate outliers. We base this selection on a filter that eliminates the brightest 20% and darkest 20% of pixels, defining the pixel brightness as its average intensity. Fourth, we calculated the spectral signature using the average reflectance spectra of the remaining 60 percent of the pixels. These four steps made it possible to extract a representative, unidimensional spectral signature of the wastewater. We provided more details on this spectral signature extraction procedure in Lechevallier *et al*.^10^.

### Processing of organic chemical data

Organic chemical data were acquired in dynamic multiple reaction monitoring (MRM) mode and subsequently analyzed with the MassHunter Quantitative Analysis Software (Version 10.1, Agilent Technologies, Inc., U.S.A.). We chose the ISTD of the analyte with the most similar retention time and measured in the same ion mode for the quantification of target analytes without a corresponding ISTD, as shown in Supplementary Table 8. We calculated matrix factors (MF) for all analytes using samples spiked with the analyte standard (Supplementary File 2, equations (2) and (3)). As shown in Supplementary Table 10, MFs ranged from 0.5 to 1.5 for most analytes and rain events. We used them to adjust the limits of quantification (LOQs) derived from the calibration curve for potential matrix effects. Supplementary Table 11 shows that the LOQ_MF corr._ was below 50 ng/L for most of the analytes and generally higher in samples from the second measurement series, when the instrument’s sensitivity was lower. The LOQ_MF corr._ of triclosan, 2,4-D, OIT, DEET, 4-&5-methylbenzotriazole, and benzotriazole was increased either due to analytical difficulties (triclosan, 2,4-D, OIT, and DEET) or contamination of the instrument (4-&5-methylbenzotriazole and benzotriazole). The LOQ_MF corr._of acesulfame, cyclamate, and caffeine ranged from 0.8 to 6.7 μg/L due to measurements in 100-fold diluted samples. In most blanks and blinds, the analytes were below LOQ_MF corr._. In some blanks measured in the second half of the measurement series, we detected only 4-&5-methylbenzotriazole, HMMM, and OIT. This suggests that highly concentrated samples contaminated the chromatographic column, resulting in limited carry-over.

Relative recoveries (RRs) of the analytes were also calculated based on the samples spiked with the analyte standard (Supplementary File 2 equation (1)). RRs were used to correct the measured concentrations of the analytes without ISTD (Supplementary Table 9). Measured concentrations in samples taken during low flow and high flow conditions were corrected with RR calculated for a spiked sample taken during low flow and high flow, respectively. Samples with ISTD were not corrected with the calculated RR because the ISTD already compensates for possible matrix effects. The RRs of most analytes were in the acceptable range from 75% to 125%. Only the RR of 4-&5-methylbenzotriazole, benzotriazole, and DEET exceeded this range, probably due to contamination of the instrument. The RRs of the target analytes in the Pharma-Mix17 were in the acceptable range or slightly elevated (up to 159%) during both measurement series (Supplementary Table 12). The slightly elevated values indicate limited carry-over due to high concentrations contaminating the column throughout the measurement series.

The field blanks were turbid and contained large particles from previous wastewater samples. As a result, we detected the analytes at high concentrations in the wastewater-stormwater mix in the field blanks (Supplementary Table 13). Depending on the substances and rain events, the concentrations detected in the field blanks ranged from 0 to 30% of those measured in the previously collected samples. This indicates that the autosampler’s performance was not perfect and that cross-contamination between samples is likely.

## Data Records

Data and codes are available in ERIC open, the Eawag Research Data Institutional Collection: 10.25678/000D3C^19^.

**Fig. 6:**
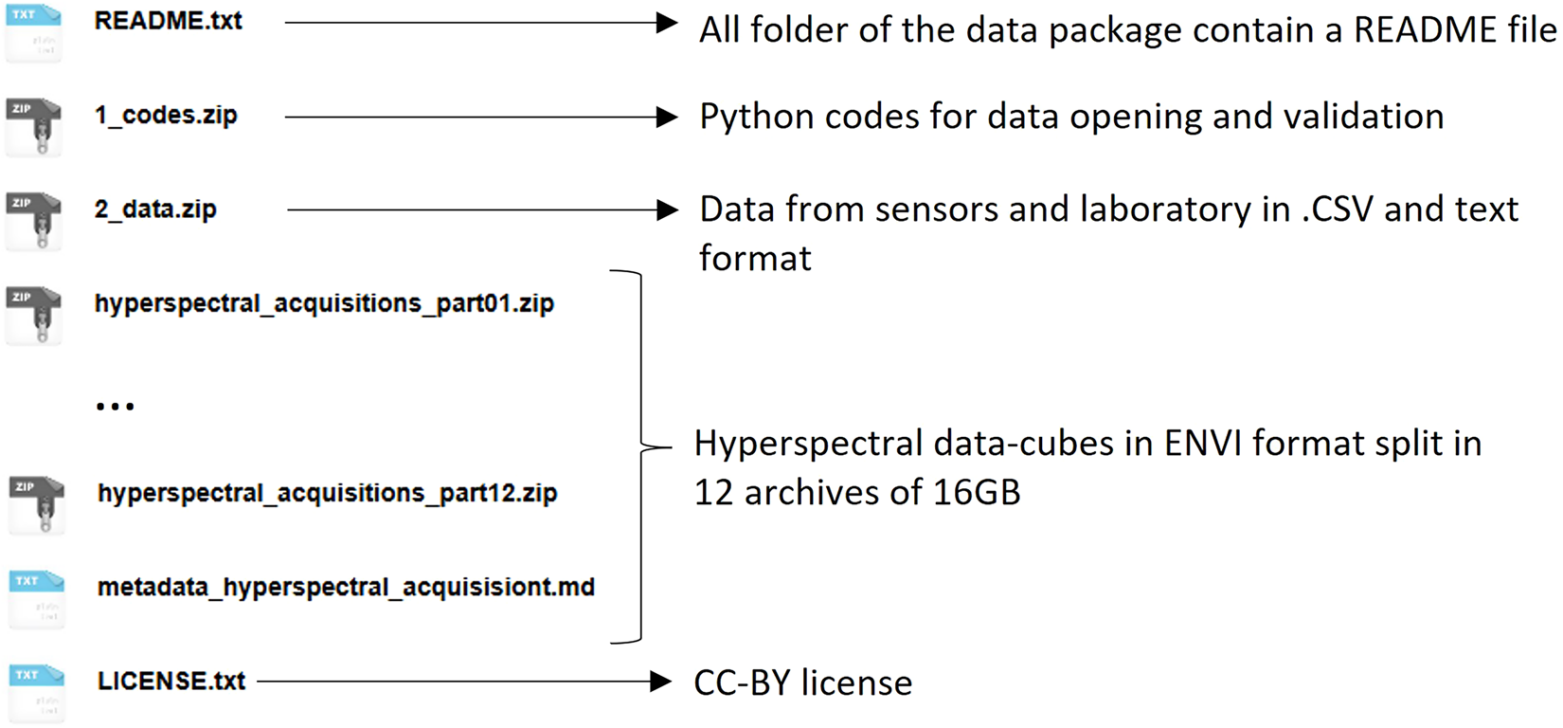
Organization of the dataset in 14 archives and readme files.

This package consists of 14 archives, as shown in Fig. 6. Each folder of the dataset contains its own metadata text file with all relevant information to open, understand, and use the data. All data are either in CSV or ENVI format, and the metadata are in text format.

The hyperspectral datacubes are stored in 12 archives of 16 GB each. Each datacube is in the ENVI format in a folder named after the timestamp of acquisition (format: YYYY-MM-DDThh.mm.ss, with YYYY: year, MM: month, DD: day, hh: hour, mm: minute, ss: second). ENVI datacubes can be opened with Python. We provided Python codes in the 2_codes.zip archive to open and preprocess the datacubes.

**Fig. 7:**
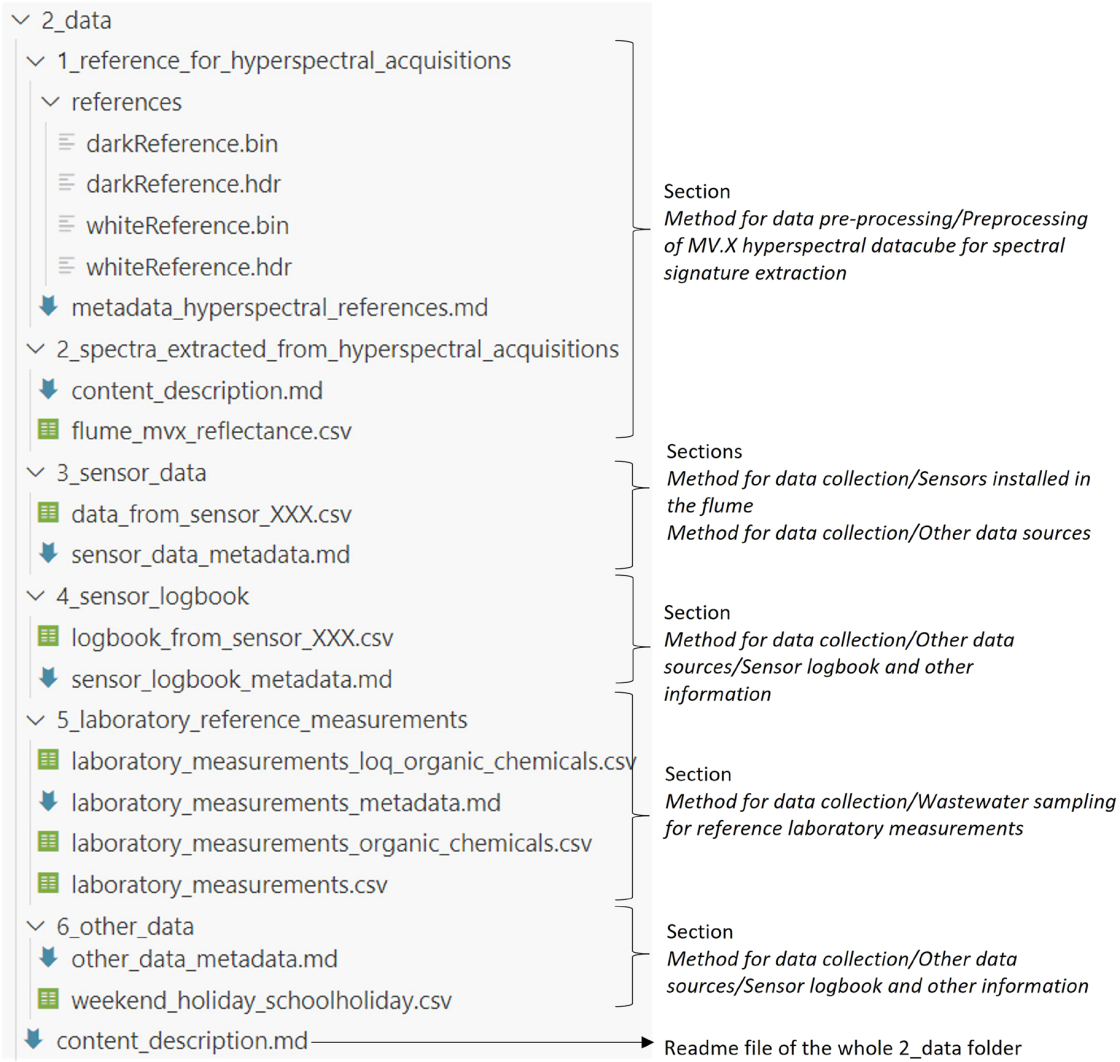
Organization of the 2_data.zip archive with the content of each of the six subfolders and the corresponding section where the data collection is described.

The remaining data, archived in 2_data.zip, are organized in 6 subfolders. Figure 7 shows the content of each subfolder and in which section relevant information regarding data collection or preprocessing can be found. Each data file name and column is named uniquely as described above.

## Technical Validation

The validated data, obtained after applying preprocessing and validation steps, includes sensor data, laboratory reference measurements, catchment precipitation, and occupancy. Out of the eight sensors operated in this experiment, six produced validated data. We considered the data from the ISEmax ion-selective electrode and E53 conductimeter invalid due to the absence of pressurized air cleaning (see below). Table 3 shows the number of valid data points for each data source.

**Table 3:** Overview of the validated data for each sensor.

Data name	Data location	Sensor	Number of valid data	Range of valid data
Catchment precipitation	Eawag campus	Pluvio2S	6,674	0.019-14.554 mm
VNIR datacubes	Flume	MV.X	5,801	N.A.
VNIR reflectance spectra	Flume	MV.X	5,544	0.5-0.25 (at 600 nm)
UV-vis absorbance spectra	Flume	Spectrolyser	118,892	16.9-678 m^-1^ (at 255 nm)
UV-vis absorbance spectra	Flume	ISA	114,646	0.02-1 (at 254 nm)
Raw ammonium, pH, Temperature	Flume	ISEmax	Data not valid	Data not valid
Temperature	Flume	Spectrolyser	118,657	15.6-23.6°C
Turbidity	Flume	Turbimax	89,865	23.4-857.4 NTU
Electrical conductivity	Eawag hall	E53	Data not valid	Data not valid
Sewer flow	Eawag hall	Nivus CS2	197,313	67.6-876.0 L/s
Sewer level	Eawag hall	Nivus i3	201,647	106.2-998.4 mm
Occupancy	Telecom data	N.A.	5,542	9,666-21,225
Reference measurements	Laboratory	N.A.	533	N.A.
Organic chemicals	Laboratory (LC-HRMS/MS)	N.A.	86	N.A.

While two sensors did not pass the validation criteria, the remaining ones worked for almost the whole measurement campaign with an acquisition every two minutes.

### Correlation analysis for data validation

In addition, we used Pearson correlations for consistency checks. This was only applicable when data from two independent sources were expected to be correlated. Those correlations are present either when a variable is measured independently twice, e.g., turbidity with a sensor and in the laboratory, or when two variables are expected to be correlated due to physical processes, e.g., turbidity and TSS. This consistency check could be applied to most data, with the exception of laboratory measurements of organic chemicals, PO_4_-P and SO_4_-S, as well as sensor data from the MV.X hyperspectral camera, from the E53 EC-meter, and from the ISEmax pH-meter, for which we did not have redundant measurements. We still considered those data that did not qualify for consistency reliable, with the exception of the E53 EC-meter, which was not properly maintained during the experiment. For the other sensors that could be validated, results are presented in Table 4. Most of the consistency checks are successful with a high positive Pearson correlation (>0.8), demonstrating that the data are reliable. Only for the ISEmax sensors, the correlation is low (0.2). We discuss in the next section why we do not consider the ISEmax data as valid. We also dedicated a final section to a more detailed data validation of the spectrophotometric data.

**Table 4:** Pearson correlation between independent data sources used for consistency check.

Data source 1	Data source 2	Pearson correlation	Comment
Flow from i3 sensor	Level from cs2 sensor	0.96	Level and flow in the sewers should be correlated
Temperature from ISEmax sensor	Temperature from Spectrolyser sensor	0.99	
Absorbance from ISA sensor	Absorbance from Spectrolyser sensor	0.87	See discussion
NH4-N from ISEmax sensor	NH4-N from laboratory	0.20	
DOC from laboratory	TOC from laboratory	0.78	DOC is typically 50–80% of TOC in raw wastewater
TDN from laboratory	TN from laboratory	0.93	TDN is typically 90–100% of TN in raw wastewater
NH4-N from laboratory	TDN from laboratory	0.92	NH4-N is typically 60–80% of TDN in raw wastewater
Turbidity from Turbimax sensor	Turbidity from laboratory	0.89	
Turbidity from Turbimax sensor	TSS from laboratory	0.95	Turbidity is traditionally used as proxy for TSS
Occupancy from KidoDynamics	Holidays	Visual correlation	See discussion
Precipitation	Flow from i3 sensor	Visual correlation	See discussion

**Fig. 8:**
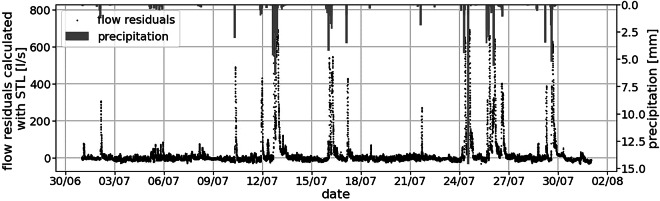
Visualization of the flow residuals and precipitation for the month of July 2023 shows that most of the flow residual peaks occur shortly after rain events.

We validated the precipitation data qualitatively by comparing them with the flow residuals extracted with STL decomposition (see the details of the calculation in Supplementary file 1). Figure 8 shows, for the month of July, that the flow residuals are around zero most of the time but are increasing shortly after each rain event. Results for the other months are similar, validating that the flow and precipitation data are coherent. We also use the delay between flow residual and precipitation to estimate that the average response time of the catchment is around 96 minutes (see details in Supplementary Figure 13). This delay is likely different for each rain event, depending on the intensity, duration, localization of the rain, antecedent dry period, and evaporation potential. In summary, for the purpose of this study, the flow, level, and precipitation data are consistent.

**Fig. 9:**
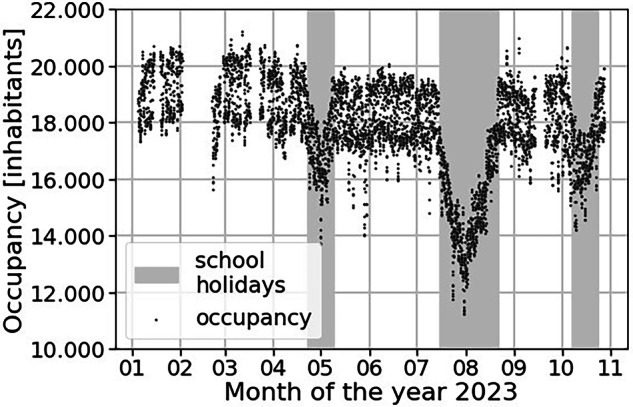
Visualization of the catchment occupancy (estimated population density) from January to November 2023, alongside the school holidays. The catchment population usually fluctuates between 18.000 and 20.000 inhabitants, except during holidays, when the occupancy drops to 16.000 and 12.000.

We also verified quantitatively the catchment occupancy data by looking at the variations during holidays (see Fig. 9). We observe that the occupancy is linearly diminishing and then increasing during the months of May, July-August, and October, corresponding to the school holidays. During the summer holidays, we observe the biggest reduction of occupancy. Overall, those results enable us to validate the occupancy data.

### Analysis of the ISEmax data reveals that they are not reliable

**Fig. 10:**
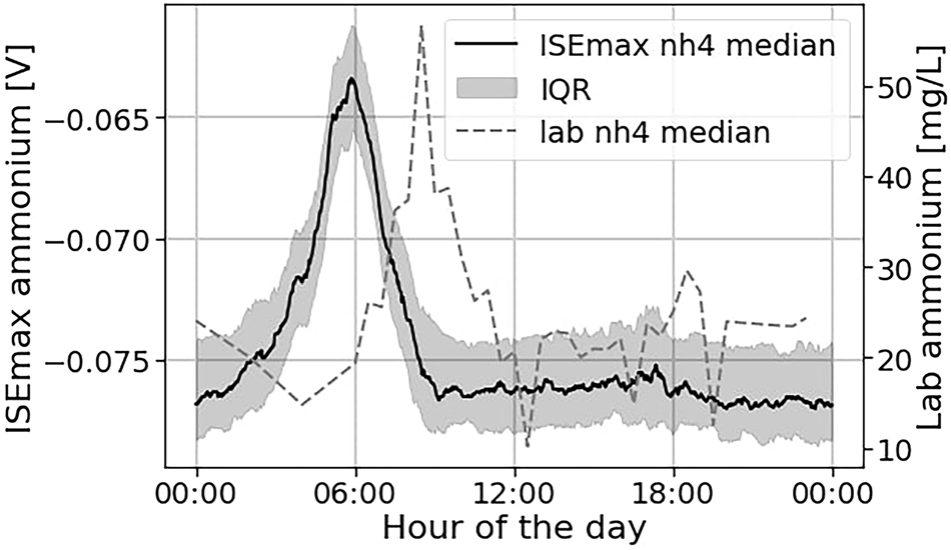
Median ammonium signal from the ISEmax ion selective electrode and median laboratory ammonium. The ISEmax signal is not following the expected trend captured with laboratory reference measurement.

As the correlation between ammonium laboratory reference measurements and ISEmax sensor data is low (see Table 4), the sensor was not considered. Moreover, as Fig. 10 illustrates, the observed daily ammonium trend is highly unusual. While the right side of the curve is coherent, with a slight evening peak around 18:00, the left side displays a wide peak starting at around 0:00 and lasting until around 9:00. This strong night peak does not reflect the expected ammonium concentration, which should first rise later in the morning when people start waking up, as verified by laboratory measurements.

To explain the discrepancies between ISEmax and laboratory ammonium, one of our hypotheses was that the ISEmax clock was experiencing a time delay of about three hours. However, we verified that the timestamps were correct by comparing the temperature readings from the ISEmax sensor with those from the scan sensor, which correlated very well (Pearson >0.99). Furthermore, even after introducing a time delay of three hours in the ISEmax data, the correlation with laboratory data remains low (Pearson: 0.53). Another hypothesis that we refuted was that the absence of automated air cleaning had a negative impact on data quality. To disprove this hypothesis, we conducted subsequent experiments with the same sensor at the same location, this time equipped with air cleaning, which revealed the same inconsistencies. Our two main explanations for the ISEmax failure are either that the sensor is simply not reliable or that nightly industrial discharges are disturbing the electrode. Ammonium-ion selective electrodes are known to be sensitive to cations such as potassium (K^+^), sodium (Na^+^), and magnesium (Mg^2+^)^20^.

Similarly to ammonium, we did not consider the pH data reliable due to the sensor not being equipped with air cleaning. Contrary to the other data signals, which followed a clear daily pattern, it was only possible to extract an approximate pattern from the pH data. Therefore, we do not recommend using the pH data except to identify relative trends.

### Validation of the spectrophotometric measurements

**Fig. 11:**
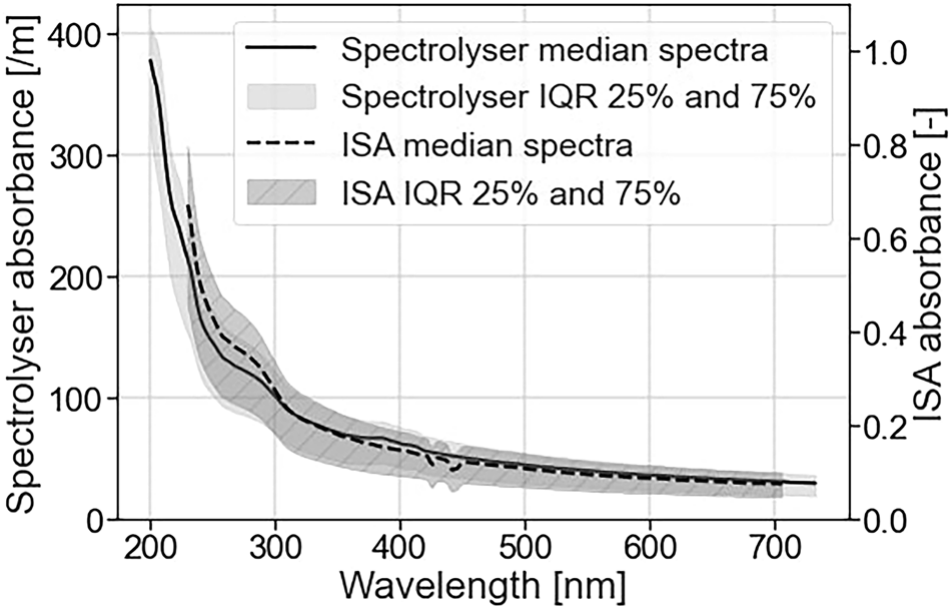
Comparison of the median Spectrolyser spectra and ISA spectra shows that despite measuring the same wastewaters, the spectra shape is not the same for each sensor.

In addition to the mean Pearson correlation of 0.87 (see Table 4), we validated absorbance spectra from the ISA and the Spectrolyser sensor by comparing the median and interquartile range (IQR) of the absorbance spectra. Both show similar global shapes, but with minor differences (as shown in Fig. 11). It is common for wastewater samples to have this absorbance profile, with high absorption at lower wavelengths and exponential decay at higher wavelengths^21^. This is because organic compounds in wastewater absorb light in the UV range. Both median spectra have a significant shoulder between 260 and 300 nm. The interquartile range (IQR) spreads consistently on each side of the median and is relatively narrow (+/− 25 [m] for Spectrolyser and 0.05 for ISA). However, there are also differences in the spectra, such as around 430 nm, where the ISA spectra show oscillations while the Spectrolyser spectra remain linear. Furthermore, due to the removal of data on both ends of the spectra, the ISA’s measurement range is narrower (230–706 nm) than the Spectrolyser’s (200–735 nm).

The discrepancies in local shape and range are due to technological differences. For the Spectrolyser, the sensor head contains the light and spectrophotometer. The ISA instruments use several meters of optical fiber cable to connect the sensor head to the analytical instruments. Because those instruments are sensitive, keeping them separate from the sensor head makes it possible to monitor in extreme conditions, such as high temperatures. However, long optical fibers absorb low ultraviolet light, which explains why we had to discard the wavelength below 230 nm.

**Fig. 12:**
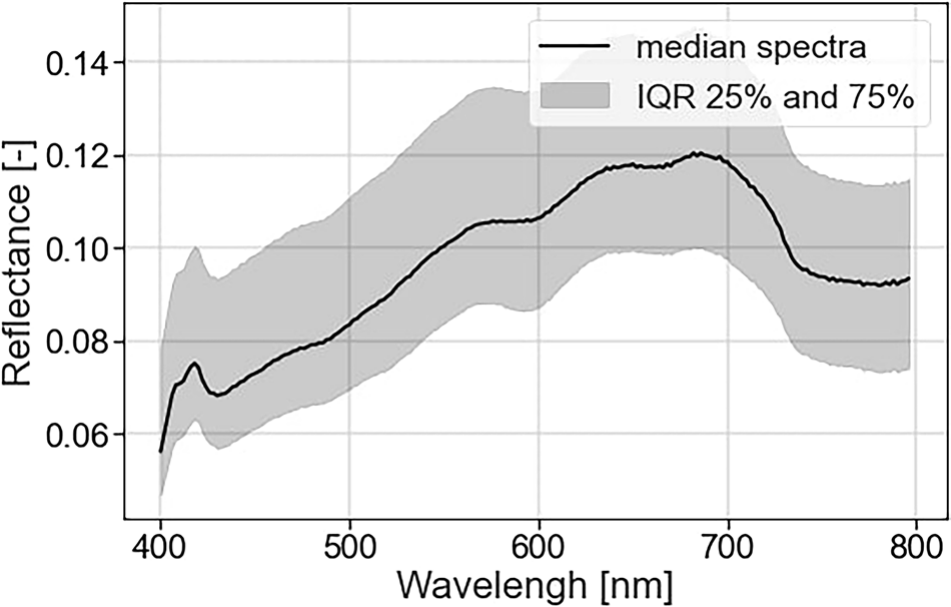
Median and 50% IQR of the reflectance spectra extracted from the wastewater hyperspectral datacubes.

The reflectance spectra display a similar shape as our previous work in the laboratory^10^ (see Fig. 12). Contrary to the absorbance spectra, the reflectance is increasing in the 400–700 range before decreasing at higher wavelengths. Higher reflectance in the region between 600 and 700 nm is expected for a brown-colored solution such as wastewater. As depicted by the IQR, the spectra are typically located +/− 20% around the median. We also see that the reflectance is complementary to the absorbance, with an increase in the 400–700 nm range while the absorbance spectra are decreasing in this range. However, the relationship between absorbance and reflectance is not straightforward. Both absorbance and reflectance are the consequence of two physical mechanisms between light and wastewater constituents: absorption and scattering. Typically, light scattering drives reflectance, while absorbance primarily measures light absorption^21,22^. Therefore, both are relevant to measure and can provide different information about the wastewater constituents.

## Supplementary information

Supplementary file 1

Supplementary file 2

Supplementary figures

Supplementary tables

## Data Availability

Python codes are available in the same ERIC open repository as the data at this link: 10.25678/000D3C^19^. We included codes to open the hyperspectral data cubes and codes to open all the other CSV files shared in this dataset.
