# Supplementary material for: Dataset on wastewater quality monitoring with adsorption and reflectance spectrometry in the UV-vis range
Source: Sci Data. 2025 Jul 25;12:1296. doi: 10.1038/s41597-025-05459-x (PMC12297409; doi:10.1038/s41597-025-05459-x)
Supplement: Supplementary file 1 [file 41597_2025_5459_MOESM1_ESM.pdf]

Supplementary File 1: STL decomposition and calculation of wet weather samples

STL Decomposition

This methodology was developed to analyze trends and seasonality in time series. Detailed methods can be found here:

> *Cleveland, R. B., Cleveland, W. S., & Terpenning, I. (1990). STL: A Seasonal-Trend Decomposition Procedure Based on Loess. Journal of Official Statistics, 6(1), 3.*

We used this method to analyze:

- Trend: the trend is calculated by taking the median value of the variable over one week (or one day), and then subtracting that value from the data. We decided to use the median value instead of the average in order to minimize the influence of exceptional events such as rain or industrial discharges in the trend. The trend is useful to identify potential sensor drifts.
- Seasonality: the data are grouped by their “week-time” (or daytime), calculated in minute. For each “week-time”, the median value is calculated. Again, the median was used to minimize the impact of exceptional events.
- Residuals: they are calculated by subtracting the corresponding weekly (or daily) pattern value from the de-trended data. Since the trend and the seasonality were based on the median value, exceptional occurrences such as rain or industrial discharges are visible in the residuals.

Using STL decomposition to estimate wet weather samples

We used the residuals from the STL decomposition of the sewer flow to estimate the data points collected during wet weather. This is relevant because, during wet weather, stormwater enters the drainage system, altering the wastewater composition. We applied the STL decomposition to the available flow data, i.e., between 01/01/2023 and 31/10/2023. Figure 1 presents the results of our analysis. We already analyzed elsewhere that the flow residuals peaks correspond to rain events (section “Precipitation and sewer wastewater quantities” in Supplementary File 3). To estimate the number of wet samples in the dataset, we classified each flow data point with the following criterion: “flow residuals are higher than 20% of the maximal dry weather flow => wet weather data point”. The maximal dry weather flow being 120 L/s, the criterion becomes: “flow residuals > 24 L/s => wet weather datapoint”. We present the results of this classification in Figure 2, where we can verify the plausibility of the criterion used.

With this dry/wet weather classification, 74.4% of the data points between 08/05/2023 and 26/10/2023 are classified as dry weather. This percentage means that approximately ¼ of the data collected with sensors during the flume experiment was in wet weather. We collected 199 laboratory samples during wet weather, which corresponds to 38% of the total number of laboratory samples collected. We oversampled in wet weather because we sampled four rain events in high resolution for organic chemical analysis, corresponding to 86 samples.

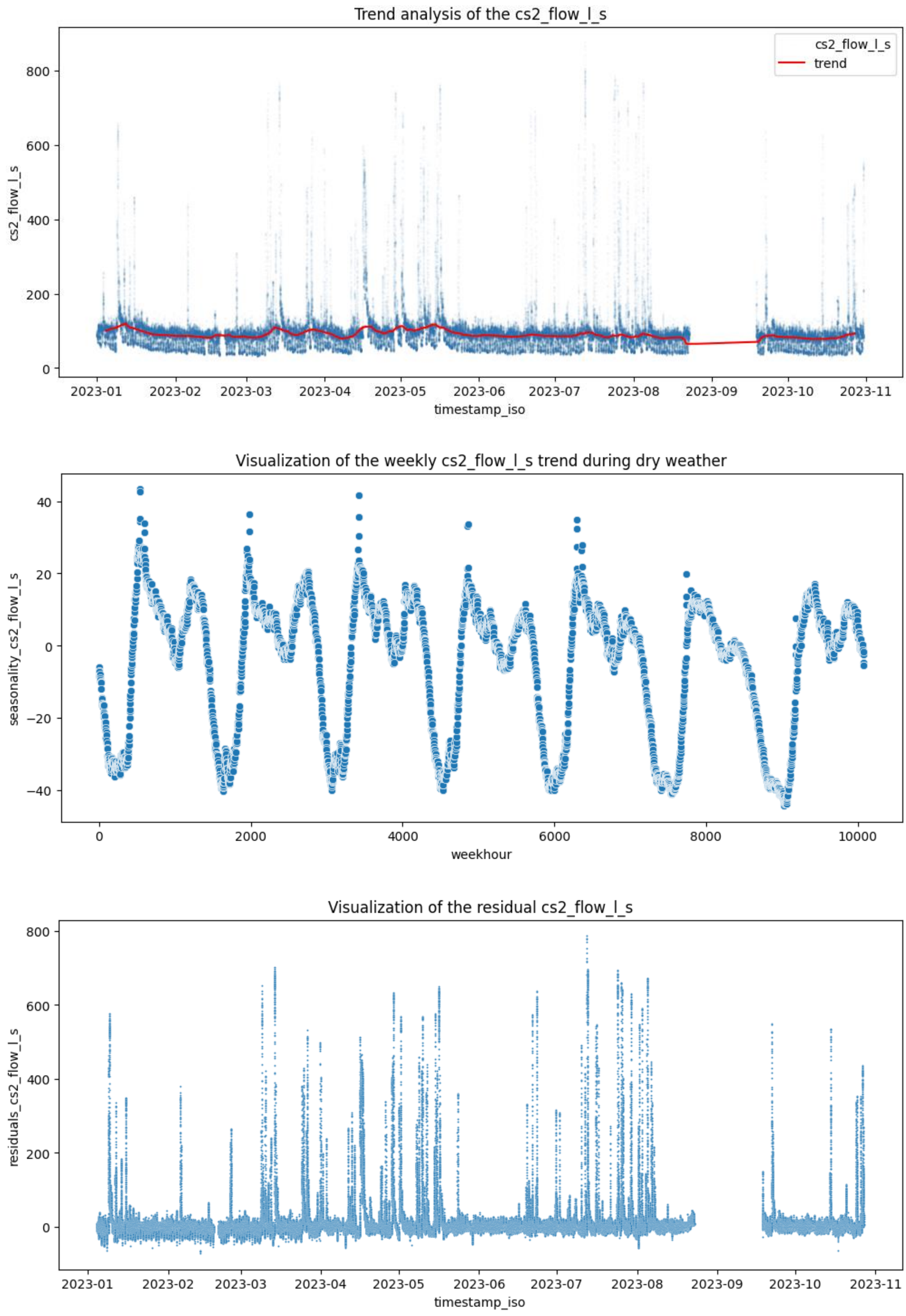

Figure 1: Trend (top), weekly seasonality (middle) and residuals (bottom) of the flow measurements in the sewer calculated with STL decomposition.

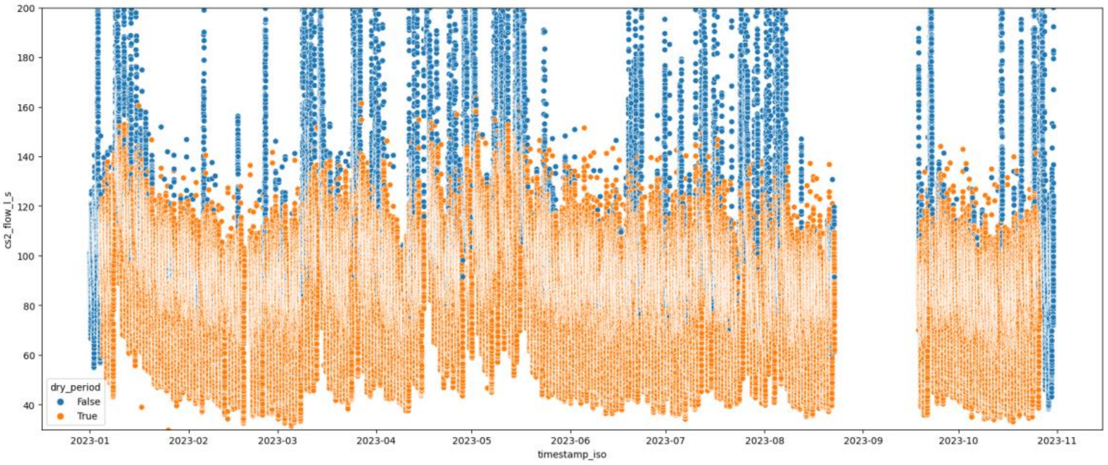
cs2_flow_l_s
timestamp_iso
dry_period
False
True
200
180
160
140
120
100
80
60
40
2023-01
2023-02
2023-03
2023-04
2023-05
2023-06
2023-07
2023-08
2023-09
2023-10
2023-11
