# Supplementary material for: Dataset on wastewater quality monitoring with adsorption and reflectance spectrometry in the UV-vis range
Source: Sci Data. 2025 Jul 25;12:1296. doi: 10.1038/s41597-025-05459-x (PMC12297409; doi:10.1038/s41597-025-05459-x)
Supplement: Supplementary file 2 [file 41597_2025_5459_MOESM2_ESM.pdf]

Supplementary file 2: LC-HRMS/MS analysis of organic chemicals

Supplementary Table 4: Organic chemicals analyzed during rain events. They were assigned to municipal (+ industrial) wastewater, stormwater or both depending on where their concentrations were expected to be highest. Abbreviations: personal care product (PCP), plant protection product (PPP). Chemical data source: ChemSpider, PubChem.

	Substance	Use	Log(K_{ow})
**Municipal (+ industrial) wastewater**	Acesulfame	Food & beverages (sweetener)	-1.33
	Caffeine	Food & beverages (coffee, tea)	-0.07
	Cyclamate	Food & beverages (sweetener)	-1.61
	Candesartan	Pharmaceutical (antihypertensive drug)	4.79
	Citalopram	Pharmaceutical (antidepressant)	1.39
	Diclofenac	Pharmaceutical (non-steroidal anti-inflammatory drug)	4.51
	Hydrochlorothiazide	Pharmaceutical (antihypertensive drug)	-0.07
	Triclosan	PCP (anti-microbial agent)	4.76
**Stormwater**	1,3-Diphenylguanidine	Road runoff (vulcanization accelerator)	2.89
	6PPD-Quinone	Road runoff (ozone protection and antioxidant in tires)	4.47
	Hexa(methoxymethyl) melamine (HMMM)	Road runoff (cross-linking agent in coatings & tires)	-0.05
	2,4-D	PPP	2.81
	Diuron	Biocide (facades) & legacy PPP	2.68
	Carbendazim	Biocide (facades) & legacy PPP	1.52
	2-methyl-4-chloro-phenoxyacetic acid (MCPA)	PPP	3.25
	Mecoprop-p	PPP & industrial chemical (flat roof)	3.13
	2-n-Octyl-4-isothiazolin-3-on (OIT)	Biocide (building materials)	2.45
**Both**	4-&5-Methylbenzotriazole	Roads, household & industry	1.71
	Benzotriazole	Roads, household & industry	1.44
	N-N-diethyl-3-methylbenzamide (DEET)	PCP (insect repellent sprays) & biocide	2.02

Chemical analysis was performed using LC-HRMS/MS. Full scan MS detection was performed with an Agilent 6495C triple quadrupole mass spectrometer with electrospray ionization. Data was acquired in dynamic MRM mode using a list of the target analytes. Samples were analyzed in two separate runs in positive and negative ionization mode. For chromatographic separation, an Acquity UPLC HSS T3 column (3×100 mm, 1.8 µm particle size) and an Acquity UPLC HSS T3 VanGuard precolumn (2.1×5 mm, 1.8 µm particle size) (Waters Corporation) were used. The injection volume was 100 µL. The HPLC gradient was formed by changing the mixing ratio of acidic nanopure water and acidic methanol, both solvents containing 0.1% formic acid (Supplementary Table).

Supplementary Table 5: HPLC gradient used for chromatographic separation of the target analytes on an Acquity UPLC HSS T3 column.

Time [min]	H_2O + 0.1% Formic Acid [%]	MeOH + 0.1% Formic Acid [%]	Flow [µL/min]

1.0	100	0	500
18.5	5	95	500
22.0	5	95	500
22.5	100	0	500

Supplementary Table 6: Settings for an Agilent 6495C triple quadrupole mass spectrometer.

**Parameter**	**Positive and negative mode**
**ESI settings**	
Gas temperature	250°C
Gas flow	15 L/min
Nebulizer	25 psi
Sheath gas temperature	325 °C
Sheath gas flow	12 L/min
Capillary	3500 V (positive), 3000 V (negative)
Nozzle voltage	1500 V
**QQQ settings**	
Cycle time	650 ms
Acquisition type	DynamicMRM
MRM repeats	3
Ion mode	ESI+Agilent Jet Stream

Supplementary Table 7: Acquisition method on an Agilent 6495C triple quadrupole mass spectrometer.

**Target analyte**	**ISTD**	**Precursor ion [m/z]**	**MS1 res**	**Product ion [m/z]**	**MS2 res**	**Frag-mentor [V]**	**Collision energy [V]**	**Ret. Time [min]**	**Polarity**
1,3-Diphenyl-guanidine	No	212.1	Unit	195	Unit	166	20	9.3	Pos.
1,3-Diphenyl-guanidine	No	212.1	Unit	119	Unit	166	24	9.3	Pos.
2,4-D	No	220.96	Unit	162.95	Unit	166	15	15.3	Neg.
2,4-D	No	218.96	Unit	160.96	Unit	166	15	15.3	Neg.
2,4-D	No	218.96	Unit	124.9	Unit	166	35	15.3	Neg.
2,4-D-D3	Yes	224	Unit	165.9	Unit	166	15	15.25	Neg.
2,4-D-D3	Yes	222	Unit	164	Unit	166	15	15.25	Neg.
2,4-D-D3	Yes	222	Unit	127.2	Unit	166	15	15.25	Neg.
4-&5-Methyl-benzotriazole	No	134.07	Unit	106.1	Unit	166	18	11.7	Pos.
4-&5-Methyl-benzotriazole	No	134.07	Unit	19.1	Unit	166	20	11.7	Pos.
6PPD-Quinone	No	299.2	Unit	215	Unit	166	16	18.2	Pos.
6PPD-Quinone	No	299.2	Unit	187	Unit	166	32	18.2	Pos.
6PPD-Quinone-D5	Yes	304.2	Unit	220	Unit	166	16	18.2	Pos.
6PPD-Quinone-D5	Yes	304.2	Unit	192	Unit	166	32	18.2	Pos.
Acesulfame	No	162	Unit	82.1	Unit	166	16	4.7	Neg.

Acesulfame	No	162	Unit	78	Unit	166	32	4.7	Neg.
Acesulfame-D4	Yes	166.01	Unit	86.2	Unit	166	12	4.7	Neg.
Acesulfame-D4	Yes	166.01	Unit	78	Unit	166	40	4.7	Neg.
Amisulpride	No	370.18	Unit	242	Unit	166	30	8.4	Pos.
Amisulpride	No	370.18	Unit	112.1	Unit	166	34	8.4	Pos.
Amisulpride-D5	Yes	375.21	Unit	242	Unit	166	34	8.4	Pos.
Amisulpride-D5	Yes	375.21	Unit	117.1	Unit	166	34	8.4	Pos.
Benzotriazole	No	120.06	Unit	92.1	Unit	166	16	9.5	Pos.
Benzotriazole	No	120.06	Unit	65.2	Unit	166	20	9.5	Pos.
Benzotriazole	No	120.06	Unit	39.2	Unit	166	44	9.5	Pos.
Benzotriazole-D4	No	124.08	Unit	96.1	Unit	166	18	9.5	Pos.
Benzotriazole-D4	Yes	124.08	Unit	69.1	Unit	166	26	9.5	Pos.
Benzotriazole-D4	Yes	124.08	Unit	41.2	Unit	166	52	9.5	Pos.
Caffeine	No	195.1	Unit	138	Unit	166	20	9.01	Pos.
Caffeine	No	195.1	Unit	110	Unit	166	30	9.01	Pos.
Candesartan	No	441.17	Unit	263.1	Unit	166	12	15.7	Pos.
Candesartan	No	441.17	Unit	235.2	Unit	166	24	15.7	Pos.
Candesartan-D5	Yes	446.2	Unit	268.1	Unit	166	16	15.7	Pos.
Candesartan-D5	Yes	446.2	Unit	240.1	Unit	166	24	15.7	Pos.
Carbendazim	No	192.1	Unit	160.1	Unit	166	16	8.08	Pos.
Carbendazim	No	192.1	Unit	132.1	Unit	166	32	8.08	Pos.
Carbendazim-D4	Yes	196.1	Unit	164.1	Unit	166	24	8.08	Pos.
Carbendazim-D4	Yes	196.1	Unit	136.2	Unit	166	36	8.08	Pos.
Citalopram	No	325.17	Unit	262	Unit	166	18	12.7	Pos.
Citalopram	No	325.17	Unit	108.8	Unit	166	38	12.7	Pos.
Cyclamate	No	178.1	Unit	96	Unit	166	18	7.7	Neg.
Cyclamate	No	178.1	Unit	80	Unit	166	36	7.7	Neg.
Cyclamate-D11	Yes	189.1	Unit	80	Unit	166	40	7.7	Neg.
DEET	No	192.3	Unit	119.1	Unit	166	16	15.02	Pos.
DEET	No	192.3	Unit	91	Unit	166	40	15.02	Pos.
DEET-D10	Yes	202.2	Unit	119.1	Unit	166	16	15.02	Pos.
DEET-D10	Yes	202.2	Unit	91	Unit	166	40	15.02	Pos.
Diclofenac	Yes	296	Unit	249.9	Unit	166	10	17.7	Pos.
Diclofenac	Yes	296	Unit	215	Unit	166	20	17.7	Pos.
Diclofenac	Yes	296	Unit	214.1	Unit	166	30	17.7	Pos.
Diclofenac-D4	No	300.05	Unit	254	Unit	166	10	17.7	Pos.
Diclofenac-D4	No	300.05	Unit	219.1	Unit	166	20	17.7	Pos.
Diclofenac-D4	No	300.05	Unit	218	Unit	166	34	17.7	Pos.
Diuron	No	233.03	Unit	72.1	Unit	166	20	15.4	Pos.
Diuron	No	233.03	Unit	46.1	Unit	166	16	15.4	Pos.
Diuron-D6	Yes	239.06	Unit	78.1	Unit	166	40	15.2	Pos.
Diuron-D6	Yes	239.06	Unit	52.4	Unit	166	20	15.2	Pos.
HMMM	No	391.2	Unit	207	Unit	166	20	14.8	Pos.
HMMM	No	391.2	Unit	177	Unit	166	32	14.8	Pos.

Hydrochlorothiazide	No	295.9	Unit	269	Unit	166	18	6.7	Neg.
Hydrochlorothiazide	No	295.9	Unit	204.9	Unit	166	22	6.7	Neg.
Hydrochlorothiazide-13C,D2	Yes	298.97	Unit	269.8	Unit	166	18	6.7	Neg.
Hydrochlorothiazide-13C,D2	Yes	298.97	Unit	206	Unit	166	26	6.7	Neg.
MCPA	No	201.01	Unit	143	Unit	166	15	15.5	Neg.
MCPA	No	199.02	Unit	141.01	Unit	166	15	15.5	Neg.
MCPA-D3	Yes	204	Unit	146	Unit	166	15	15.5	Neg.
MCPA-D3	Yes	202.01	Unit	144	Unit	166	15	15.5	Neg.
Mecoprop_p	No	215	Unit	143.1	Unit	166	8	16.5	Neg.
Mecoprop_p	No	213	Unit	141	Unit	166	12	16.5	Neg.
Mecoprop_p-D6	Yes	221.1	Unit	149.2	Unit	166	8	16.5	Neg.
Mecoprop_p-D6	Yes	219.1	Unit	147.3	Unit	166	12	16.5	Neg.
Metolachlor-D6	Yes	290.18	Unit	258.2	Unit	166	8	17.5	Pos.
Metolachlor-D6	Yes	290.18	Unit	182.2	Unit	166	8	17.5	Pos.
OIT	No	214.1	Unit	102	Unit	166	16	17.2	Pos.
OIT	No	214.1	Unit	71.1	Unit	166	16	17.2	Pos.
Triclosan	No	286.94	Unit	142.1	Unit	166	32	18.81	Neg.
Triclosan	No	286.94	Unit	35.2	Unit	166	20	18.81	Neg.
Venlafaxine-D6	Yes	266.3	Unit	266.3	Unit	166	10	12.1	Pos.
Venlafaxine-D6	Yes	121	Unit	121	Unit	166	30	12.1	Pos.

Targeted organic chemicals

Supplementary Table 8: List with all analyzed organic chemicals, their InChiKey for identification, the observed level of quantification (LOQ) and used isotope labelled standard used for quantification. The LOQ was determined from the calibration curve as the lowest concentration at which the signal-to-noise ratio was greater than ten, and the estimated concentration corresponded to the true concentration. As the substances were measured in two series, two LOQs are given. The first LOQ is valid for samples taken on the 21.06.23 and 24.10.23, and the second LOQ is for samples taken on the 14.10.23 and 20.10.23 and Acesulfame,

Caffeine and Cylamate. PCP: personal care product, Pharma: Pharmaceutical, PPP: Plant protection product.

Group	Substance	Use	InChiKey	LOQ [ng/L]	Isotope labelled standard
Municipal waste-water	Acesulfame	Food & beverages	YGCFIWIQZPHFLU-UHFFAOYSA-N	25<br>10	Acesulfame-D
	Caffeine	Food & beverages	RYYVLZVUVIJVGH-UHFFFAOYSA-N	50<br>50	Benzotriazole-D4
	Cyclamate	Food & beverages	HCAJEUSONLESMK-UHFFFAOYSA-N	25<br>25	Hydrochlorothiazide-13C,D2
	Candesartan	Pharma	HTQMVQVXFRQIKW-UHFFFAOYSA-N	5<br>25	Candesartan-D5
	Citalopram	Pharma	WSEQXVZVJXJVFP-UHFFFAOYSA-N	5<br>25	Venlafaxine-D6
	Diclofenac	Pharma	DCOPUUMXTXDBNB-UHFFFAOYSA-N	5<br>25	Diclofenac-D4
	Hydrochlorothiazide	Pharma	JZUFKLXOESDKRF-UHFFFAOYSA-N	5<br>25	Hydrochlorothiazide-13C,D2
	Triclosan	PCP	XEFQLINVKFYRCS-UHFFFAOYSA-N	50<br>75	Mecoprop-D6
Storm-water	1,3-Diphenyl-guanidine	Road runoff	OWRCNXZUPFZXOS-UHFFFAOYSA-N	10<br>25	Benzotriazole-D4
	6PPD-Quinone	Road runoff	UBMGKRIXKUIXFQ-UHFFFAOYSA-N	10<br>25	6PPD-Quinone-D5
	Hexa(methoxymethy l)melamine (HMMM)	Road runoff	BNCADMBVWNPPIZ-UHFFFAOYSA-N	2.5<br>5	DEET-D10
	2,4-D	PPP	OVSKIKFHRZPJSS-UHFFFAOYSA-N	25<br>50	2,4D-D3
	Carbendazim	Biocide & legacy PPP	TWFZGCMQGLPBSX-UHFFFAOYSA-N	5<br>10	Carbendazim-D4
	Diuron	Biocide & legacy PPP	XMTQQYYKAHVGBJ-UHFFFAOYSA-N	25<br>10	Diuron-D6
	2-methyl-4-chloro-phenoxyacetic acid (MCPA)	PPP	WHKUVVPPKQRRBV-UHFFFAOYSA-N	25<br>25	MCPA-D3
	Mecoprop-p	PPP & industrial chemical	WNTGYJSOUMFZEP-UHFFFAOYSA-N	25<br>10	Mecoprop-p-D6
	2-n-Octyl-4-isothiazolin-3-on (OIT)	Biocide	JPMIIZHYYWMHDT- UHFFFAOYSA-N	2.5<br>100	Metolachlor-D6
Both	4-&5-Methylbenzotriazole	Roads, household & industry	CMGDVUCDZOBDNL-UHFFFAOYSA-N (4-MBT)<br>LRUDIIUSNGCQKF-UHFFFAOYSA-N (5-MBT)	250<br>250	Venlafaxine-D6
	Benzotriazole	Roads, household & industry	QRUDEWIWKLJBPS-UHFFFAOYSA-N	25<br>10	Benzotriazole-D4
	DEET (N-N-diethyl-3-methylbenzamide)	PCP & biocide	MMOXZBCLCQITDF-UHFFFAOYSA-N	100<br>75	DEET-D10

Relative recovery

To quantify relative recovery, one sample taken during high flow conditions and one sample taken during high flow conditions of each rain event was spiked with 500 ng/L and 2500 ng/L

of the target analytes. Relative recovery was calculated as the difference between the concentration of the analyte in the spiked sample ($C_{spl,spike\ i}$ for sample i) and the concentration of the analyte in the corresponding not-spiked sample (C_{spl}), divided by the spiked concentration (C_{spike}). RRs were calculated for all spiked samples for which $C_{spl} \leq 2 * \left(C_{spl,spike\ i} - C_{spl}\right)$. For each rain event, the average RR of all spiked samples (i) was taken as the final RR:

$$RR = \frac{1}{n}\sum_{i=1}^{n} \frac{C_{spl,spike\ i} - C_{spl}}{C_{spike}} * 100 \qquad \text{(EQ.1)}$$

Supplementary Table 9: List with relative recoveries of all spiked samples. Relative recovery was calculated according to equation (1). RRs could not be calculated for analytes for which $C_{spl} \leq 2 * \left(C_{spl,spike,i} - C_{spl}\right)$ (indicated as n.a.). For samples without ISTD (Supplementary Table), the measured concentrations were corrected with these RR to account for possible matrix effects. Measured concentrations in samples taken during low flow and high flow conditions in the sewer were corrected with RR calculated based on a sample taken during low flow and high flow, respectively.

**Target analyte**	**Relative recovery [%]**							
	**21.06.23 09:37**	**21.06.23 10:40**	**14.10.23 09:44**	**14.10.23 11:04**	**20.10.23 13:00**	**20.10.23 14:00**	**24.10.23 10:40**	**24.10.23 12:00**
Acesulfame	101	104	111	101	110	107	102	96
Caffeine	104	115	108	120	132	135	114	106
Cyclamate	115	108	109	104	119	112	97	97
Candesartan	77	78	96	92	92	90	86	80
Citalopram	97	98	120	121	117	110	99	97
Diclofenac	122	106	149	223	184	176	116	120
Hydrochloro thiazide	99	102	116	118	123	109	98	98
Triclosan	96	105	109	117	112	130	114	100
1,3-Diphenylgua nidine	97	85	210	162	100	93	74	63
6PPD-Quinone	101	111	112	105	95	95	107	107
HMMM	105	107	113	99	99	100	200	117
2,4-D	110	108	113	118	111	117	112	102
Carbendazim	99	99	109	107	n.a.	202	115	105
Diuron	121	115	116	115	119	114	116	122
MCPA	104	108	106	105	104	100	106	107
Mecoprop-p	94	92	120	131	117	115	94	92
OIT	99	99	32	89	110	125	90	85
4-&5-Methylbenzo triazole	87	77	185	145	264	142	173	111
Benzotriazol e	110	91	159	n.a.	254	178	n.a.	99
DEET	168	122	156	129	168	126	121	104

Matrix factors

For substances with a structure-identical isotopic-labeled standard (Supplementary Table), the matrix factor (MF) was calculated as the average area of the ISTD in each sample ($A_{ISTD,spl\ i}$ for sample i) divided by the average area of the ISTD in the calibration standards ($A_{ISTD,cal\ j}$ for calibration standard j):

$$MF = \frac{1}{n}\sum_{i=1}^{n} \frac{A_{ISTD,spl\ i}}{\frac{1}{m}\sum_{j=1}^{m} A_{ISTD,cal\ j}} \qquad \text{(EQ.2)}$$

For analytes without ISTD, the MF of every spiked sample i was calculated as the difference between the area of the spiked sample ($A_{spl,spike\ i}$ for spiked sample i) and the area of the corresponding not-spiked sample (A_{spl}), divided by the area of the calibration standard with the spiked concentration ($A_{cal,spike}$). In this case, the MF can only be calculated if $A_{spl} \leq 2 * (A_{spl,spike\ i} - A_{spl})$. For each rain event, the average MF of all spiked samples (i) was taken as the final MF:

$$MF = \frac{1}{n}\sum_{i=1}^{n} \frac{A_{spl,spike\ i} - A_{spl}}{A_{cal,spike}} \qquad \text{(EQ.3)}$$

Supplementary Table 10: List with matrix factors MFs were calculated for the samples spiked with the reference standards according to Equations (2) and (3) for analytes with and without ISTD (Supplementary Table), respectively. MFs could not be calculated for analytes without ISTD, if $A_{spl} \leq 2 * (A_{spl,spike\ i} - A_{spl})$ (indicated as n.a.).

Target analyte	Matrix factor [-]							
	21.06.23 09:37	21.06.23 10:40	14.10.23 09:44	14.10.23 11:04	20.10.23 13:00	20.10.23 14:00	24.10.23 10:40	24.10.23 12:00
Acesulfame	1.0	0.9	1.2	0.9	1.0	1.1	1.0	1.1
Caffeine	0.7	1.0	1.1	1.0	1.5	1.2	1.2	1.0
Cyclamate	1.1	1.0	1.3	1.0	1.2	1.2	0.9	1.0
Candesartan	1.3	0.7	0.6	0.5	0.6	0.6	0.3	0.6
Citalopram	0.8	0.8	0.8	0.9	0.9	0.9	0.7	0.8
Diclofenac	0.5	0.6	1.1	1.2	1.2	0.6	0.7	0.6
Hydrochloro thiazide	0.5	0.6	0.8	0.8	0.8	0.8	0.5	0.6
Triclosan	1.4	0.7	0.7	0.7	0.6	0.7	0.5	0.6
1,3-Diphenylgua nidine	0.9	0.3	1.3	0.9	0.3	0.3	0.2	0.3
6PPD-Quinone	0.7	0.8	0.9	0.8	0.7	0.6	0.7	0.8
HMMM	1.7	0.8	0.9	0.7	0.8	0.8	n.a.	1.1
2,4-D	0.5	0.6	0.7	0.6	0.6	0.6	0.4	0.5
Carbendazim	0.5	0.6	0.7	0.7	2.7	1.0	0.5	0.6
Diuron	0.5	0.6	0.8	0.6	0.5	0.5	0.5	0.6
MCPA	0.6	0.6	0.7	0.7	0.6	0.6	0.5	0.6

Mecoprop-p	0.5	0.6	0.8	0.8	0.7	0.6	0.4	0.6
OIT	1.4	0.8	0.4	0.9	1.1	0.9	0.6	0.6
4-&5-Methylbenzotriazole	1.5	1.2	1.1	0.9	1.4	1.1	n.a.	0.9
Benzotriazole	0.5	0.6	1.0	5.8	0.6	0.6	0.5	0.7
DEET	0.6	0.8	1.1	0.8	1.1	0.8	0.7	0.9

Limits of detection (LOQs)

Limits of detection (LOQ) were first determined based on the calibration points. For each analyte, the LOQ was determined from the calibration curve as the lowest concentration at which the signal-to-noise ratio was greater than ten, and the estimated concentration corresponded to the true concentration. In a next step, the LOQs were corrected with the matrix factors (Table 7) according to $LOQ_{MF\ corr.} = LOQ/MF$. LOQs of Acesulfame, Caffeine and Cyclamate were additionally multiplied with their dilution factor. When no MF could be calculated, the LOQs could not be corrected (indicated as n.a.). As we calculated two matrix factors for each rain event, there are two LOQsMF corr.. For all samples, the higher LOQMF corr. (black) was taken as cutoff.

Supplementary Table 11: List with limits of detection corrected with the respective matrix factors (Supplementary Table). n.a. indicates that no MF could be calculated and thus the $LOQ_{MF\ corr.}$ not corrected. The higher $LOQ_{MF\ corr.}$ of each rain event is colored black.

**Target analyte**	**LOQ matrix factor corrected [ng/L]**							
	**21.06.23 09:37**	**21.06.23 10:40**	**14.10.23 09:44**	**14.10.23 11:04**	**20.10.23 13:00**	**20.10.23 14:00**	**24.10.23 10:40**	**24.10.23 12:00**
Acesulfame	876	883	846	1016	795	891	882	891
Caffeine	6684	5032	4756	5083	3342	4234	4142	5122
Cyclamate	2370	2611	1948	2449	2129	2163	2646	2402
Candesartan	7	6	37	35	36	38	7	6
Citalopram	4	8	39	46	39	42	15	9
Diclofenac	10	8	32	33	33	41	7	9
Hydrochlorothiazide	9	8	31	33	35	34	9	8
Triclosan	35	76	104	107	118	107	103	82
1,3-Diphenylguanidine	11	34	19	27	80	90	43	38
6PPD-Quinone	14	12	30	31	35	38	14	13
HMMM	1	3	6	7	6	6	n.a.	2
2,4-D	50	45	80	93	94	101	60	48
Carbendazim	10	9	15	18	22	21	11	8
Diuron	46	39	16	15	16	16	48	41
MCPA	44	43	35	39	38	40	47	45
Mecoprop-p	52	41	14	16	16	18	59	42
OIT	2	3	277	106	89	110	4	4

4-&5-Methylbenzotriazole	165	201	230	283	178	224	n.a.	263
Benzotriazole	52	39	14	15	21	26	52	37
DEET	154	126	105	111	109	114	149	117

Quality control

Supplementary Table 12: List with measured concentrations and relative recoveries of the Pharma-Mix17. For quality control, the Pharma-Mix17 (Neochema GmbH) was measured at the beginning and the end of each measurement series. Relative recovery was calculated according to equation (1) for the average concentration measured.

Target analyte	Expected concentration [ng/L]	Measured concentration [ng/L]				Average relative recovery [%]	
		1st measurement series		2nd measurement series		1st measurement series	2nd measurement series
		1st measurement	2nd measurement	1st measurement	2nd measurement		
4-&5-Methyl-benzotriazole	1000	1198	1109	1293	1283	115	129
Acesulfame	1000	1175	1215	1218	1263	120	124
Benzotriazole	1000	1601	1587	1507	1568	159	154
Candesartan	200	219	217	233	232	105	116
Citalopram	200	307	268	245	221	150	116
Diclofenac	200	247	241	243	240	119	121
Hydrochloro-thiazide	200	254	256	256	243	126	125
Mecoprop-p	200	237	258	257	256	119	128

After the rain events on 14.10.23 and 24.10.23, nanopure water was sampled from a bottle using the same sampling technique as for the wastewater samples taken before (grab sampling on 14.10.23, and time-weighted composite sampling on 24.10.23).

Supplementary Table 13: Measured concentrations of target analytes in field blinds. As field blinds nanopure water was sampled like wastewater.

Target analyte	Concentration [ng/L]	
	14.10.23 11:24 (FB)	24.10.23 14:40 (FB)
Acesulfame	2456	2761
Caffeine	8230	13251
Cyclamate	6823	4335
Candesartan	74	42

Citalopram	<LOQ	<LOQ
Diclofenac	380	35
Hydrochlorothiazide	80	31
Triclosan	<LOQ	<LOQ
1,3-Diphenylguanidine	118	<LOQ
6PPD-Quinone	<LOQ	<LOQ
HMMM	40	46
2,4-D	<LOQ	<LOQ
Carbendazim	<LOQ	<LOQ
Diuron	35	<LOQ
MCPA	<LOQ	<LOQ
Mecoprop-p	93	<LOQ
OIT	<LOQ	5
4-&5-Methylbenzotriazole	1148	657
Benzotriazole	397	860
DEET	144	<LOQ
