# Supplementary material for: Dataset on wastewater quality monitoring with adsorption and reflectance spectrometry in the UV-vis range
Source: Sci Data. 2025 Jul 25;12:1296. doi: 10.1038/s41597-025-05459-x (PMC12297409; doi:10.1038/s41597-025-05459-x)
Supplement: Supplementary file 3 — Supplementary figures [file 41597_2025_5459_MOESM3_ESM.pdf]

Supplementary Figures

Supplementary Figure 1	Map of the catchment including the sewer network, type and sub-catchment characteristics (source: swisstopo).
Supplementary Figure 2	Daily flow patterns during working and non-working days have a clear distinctive pattern.
Supplementary Figure 3	Flume geometry from the side and from the top.
Supplementary Figure 4	Flume channel dimensions.
Supplementary Figure 5	Picture of the flume channel from above.
Supplementary Figure 6	Picture showing the flume channel covered with an isolating black cover, and the inlet chamber (grey cylinder in the right of the picture).
Supplementary Figure 7	Flume inlet chamber with the ISEmax sensor.
Supplementary Figure 8	Overview of the flume stops due to cleaning and other problems.
Supplementary Figure 9	Picture of the installation of the ISA sensor, view from above.
Supplementary Figure 10	Sampling location for laboratory analysis.
Supplementary Figure 11	Temporal repartition of the samples during the flume experiment, depending on the analyzed indicator.
Supplementary Figure 12	Overview of the number of samples per daytime.
Supplementary Figure 13	Pearson correlation between precipitation and delayed flow for different time delays show that the maximum correlation is 0.51, reached for a time delay of 96 minutes, corresponding to an estimation of average catchment response time.

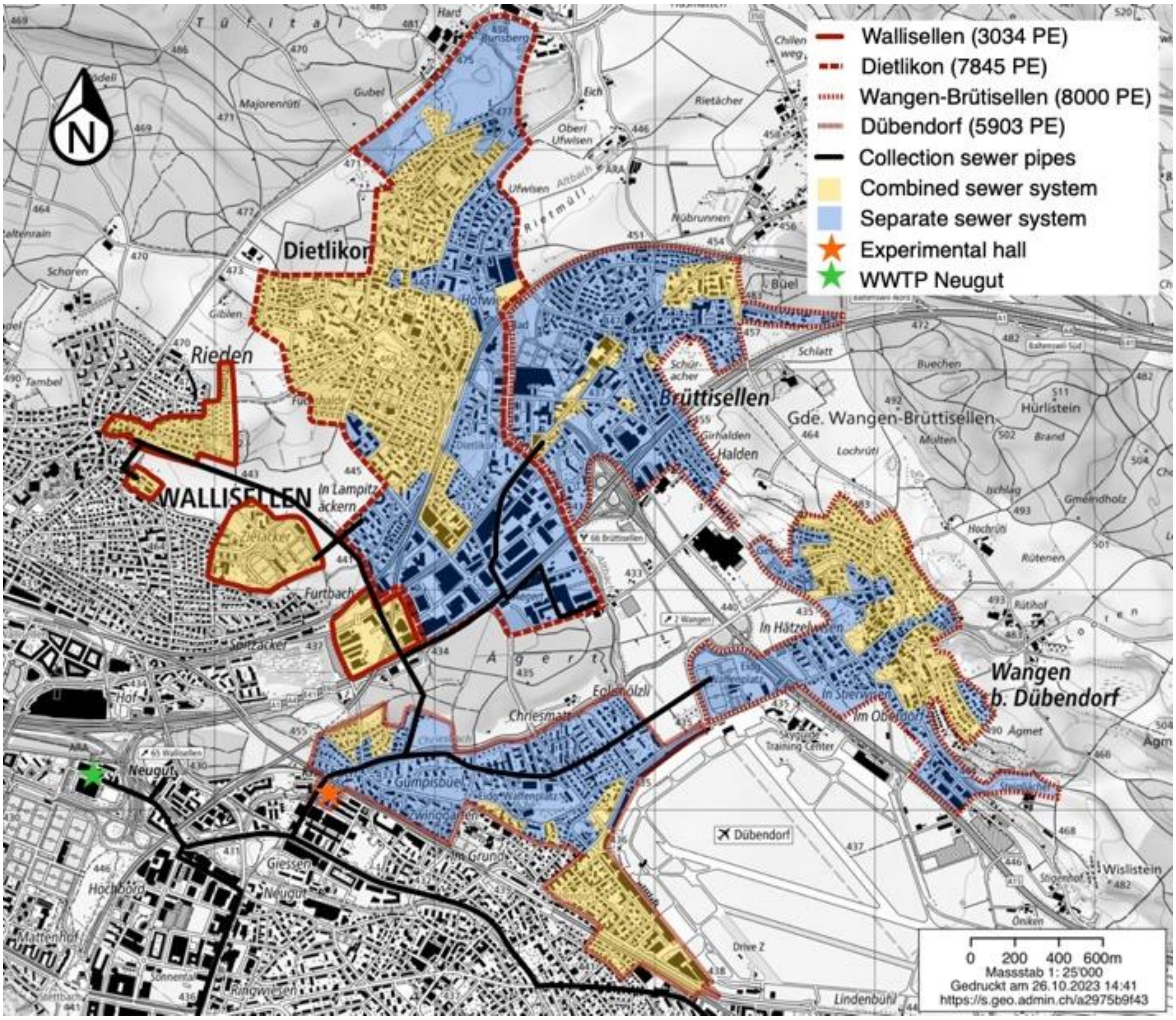

Supplementary Figure 1: Map of the catchment including the sewer network, type and sub-catchment characteristics (source: swisstopo).

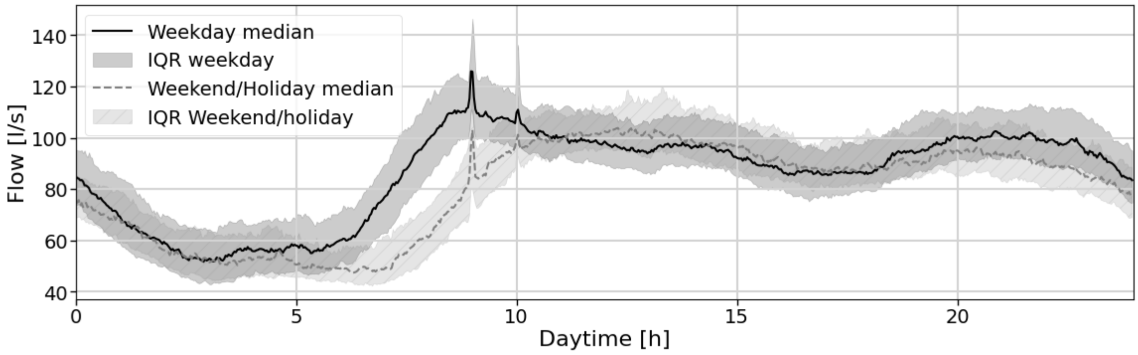

Supplementary Figure 2: Daily flow patterns during working and non-working days have a clear distinctive pattern. During workdays, an earlier and more pronounced peak is visible, and the evening peak is higher. During non-working days, the morning peak is shifted, and the overall flow is lower, showing that more people are leaving the catchment.

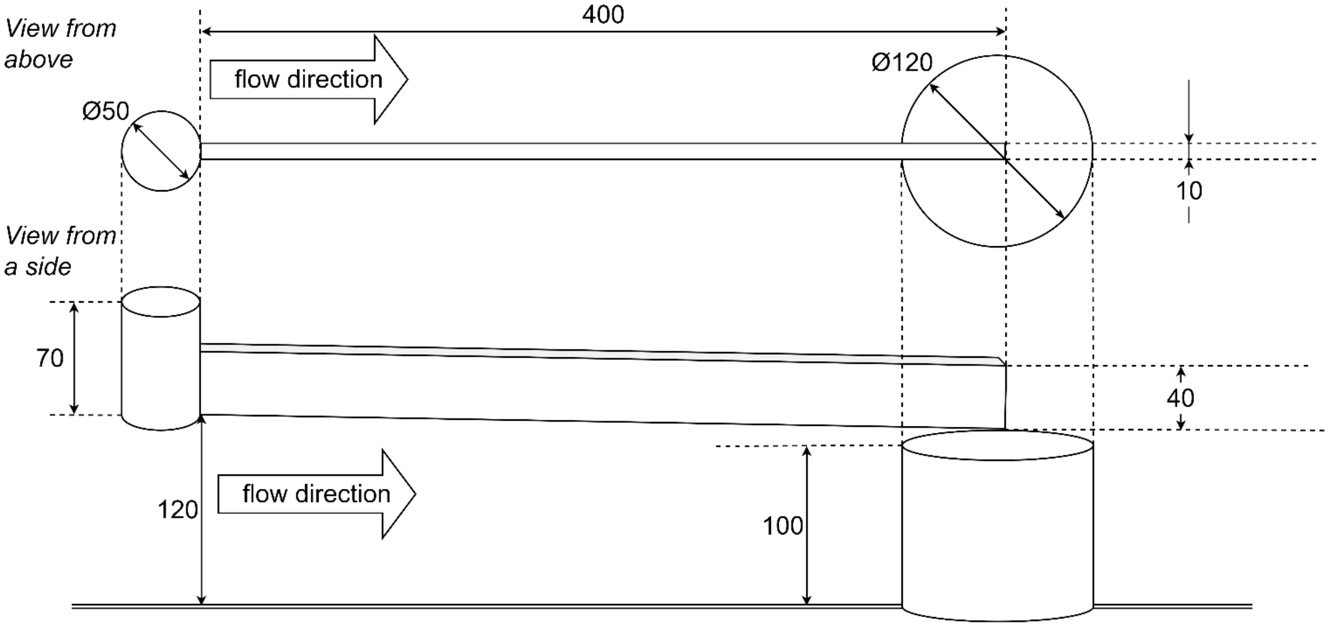

Supplementary Figure 3: Flume geometry from the side and from the top.

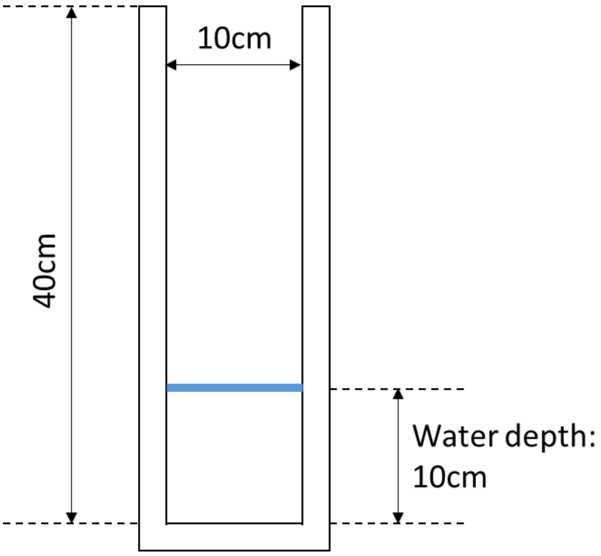

Supplementary Figure 4: Flume channel dimensions.

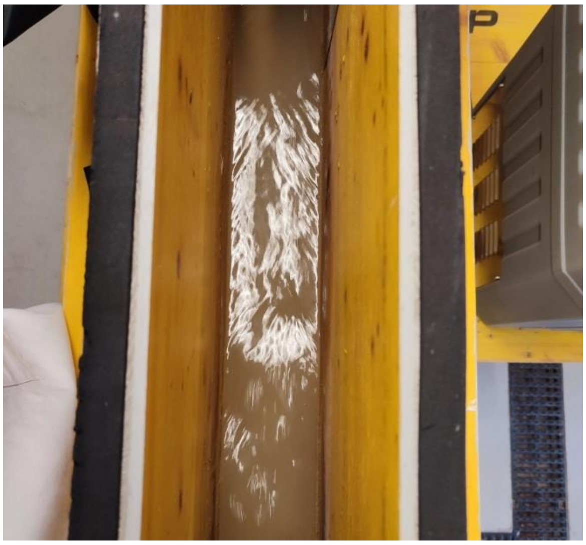

Supplementary Figure 5: Picture of the flume channel from above.

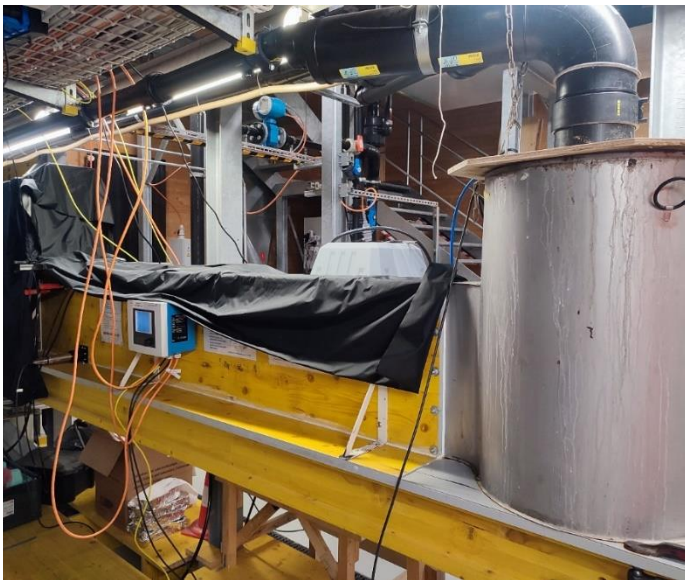

Supplementary Figure 6: Picture showing the flume channel covered with an isolating black cover, and the inlet chamber (grey cylinder in the right of the picture).

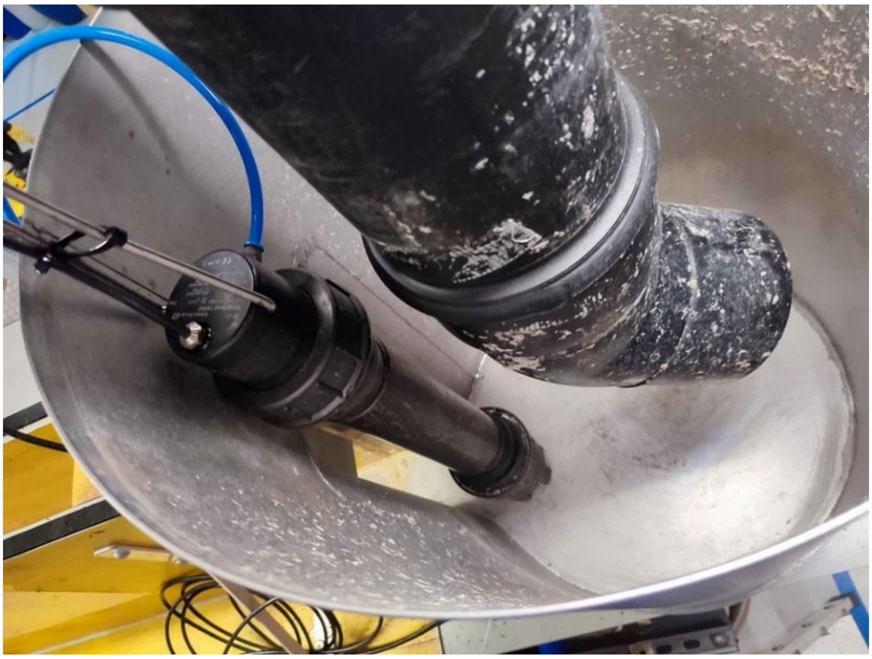

Supplementary Figure 7: Flume inlet chamber with the ISEmax sensor.

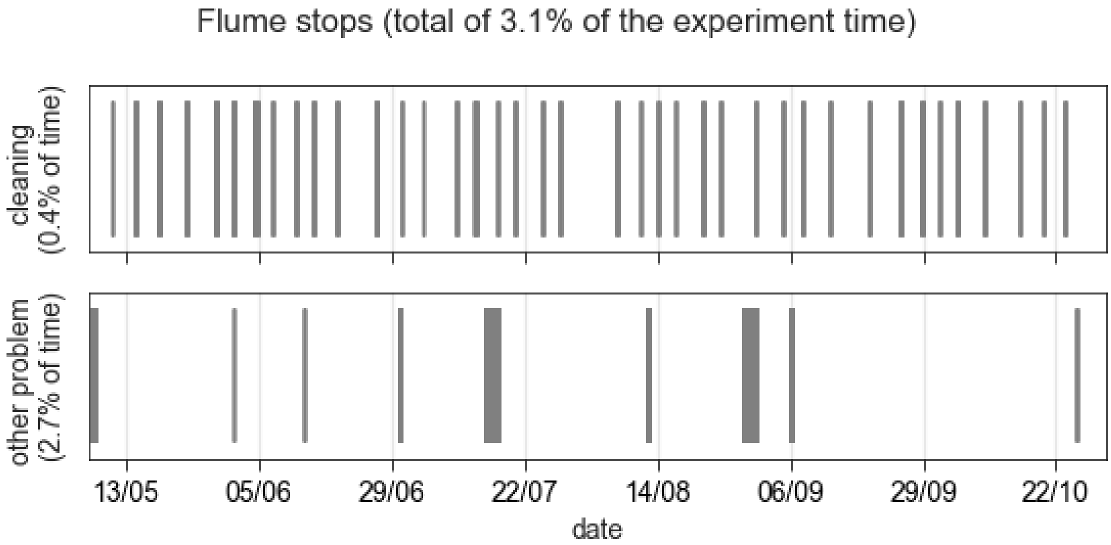

Supplementary Figure 8: Overview of the flume stops due to cleaning and other problems.

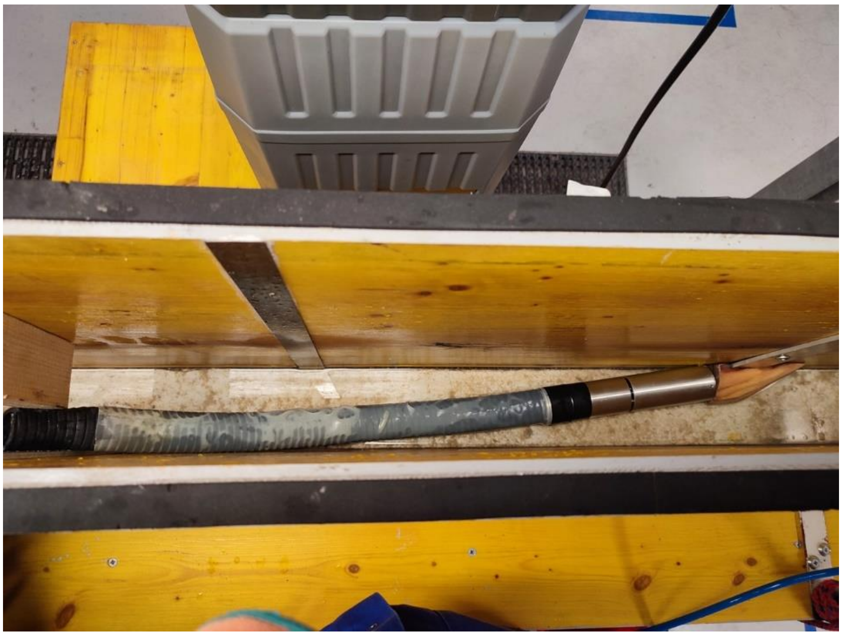

Supplementary Figure 9: Picture of the installation of the ISA sensor, view from above.

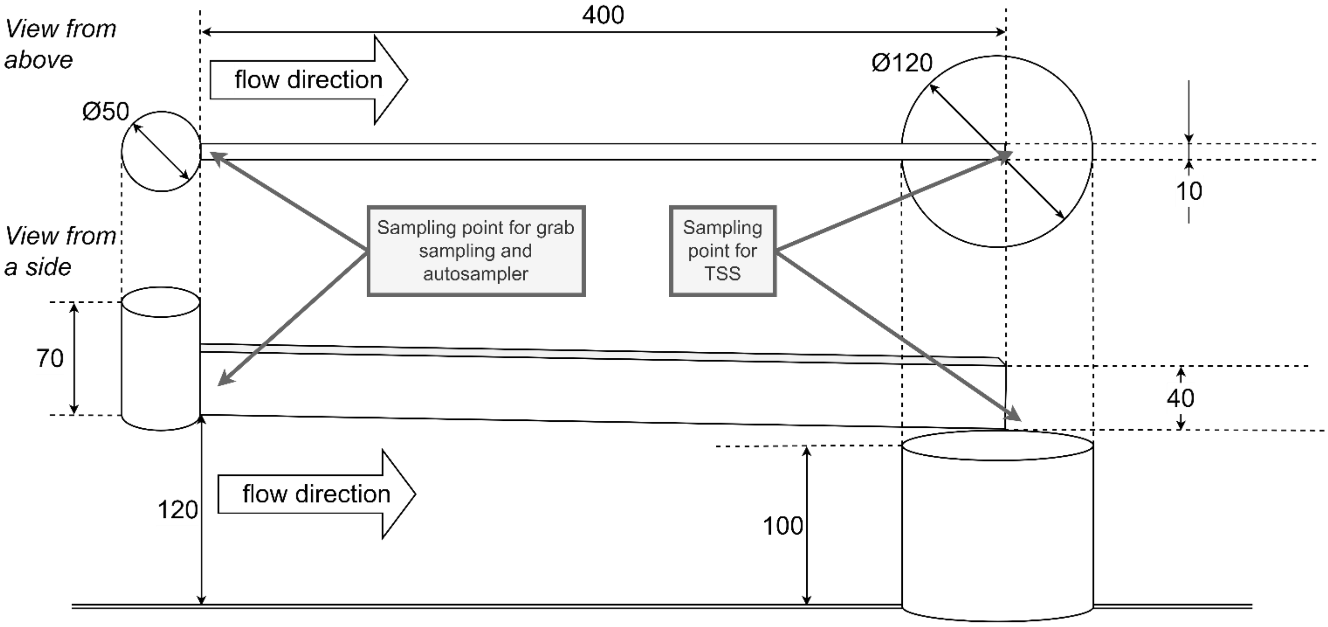

Supplementary Figure 10: Sampling location for laboratory analysis.

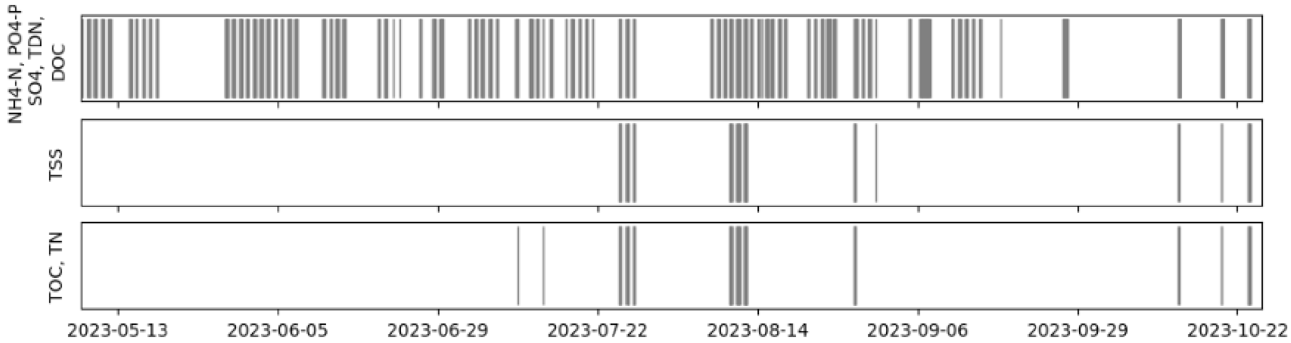

Supplementary Figure 11: Temporal repartition of the samples during the flume experiment, depending on the analyzed indicator.

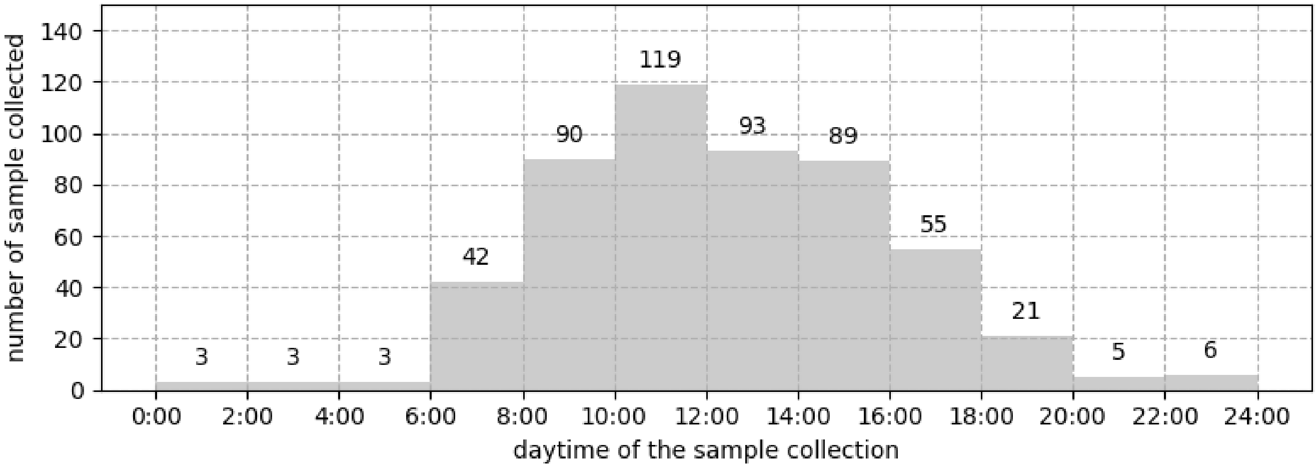

Supplementary Figure 12: Overview of the number of samples per daytime.

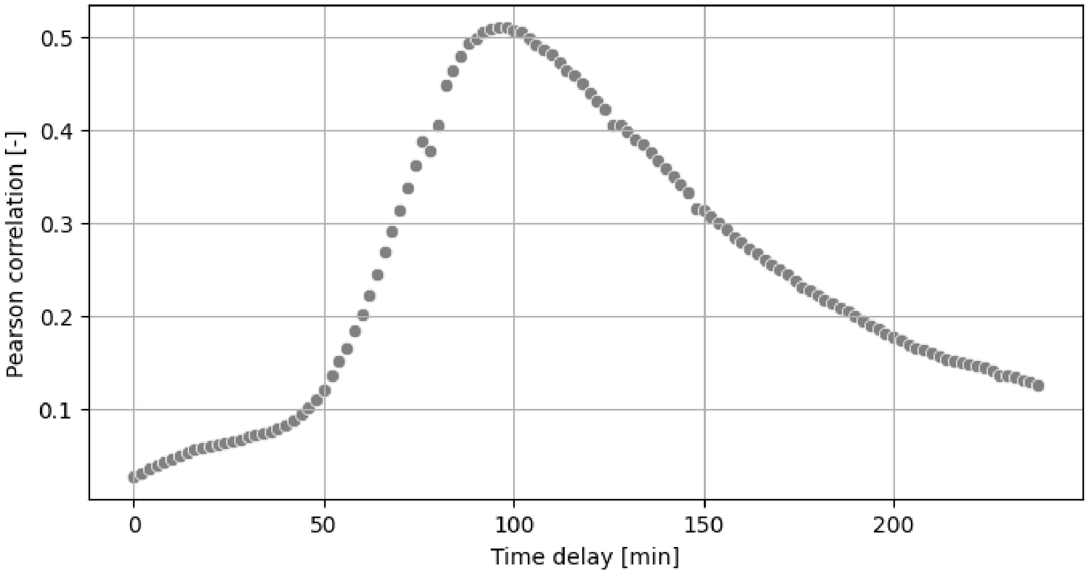

Supplementary Figure 13: Pearson correlation between precipitation and delayed flow for different time delays show that the maximum correlation is 0.51, reached for a time delay of 96 minutes, corresponding to an estimation of average catchment response time.
