# Supplementary material for: Dataset on wastewater quality monitoring with adsorption and reflectance spectrometry in the UV-vis range
Source: Sci Data. 2025 Jul 25;12:1296. doi: 10.1038/s41597-025-05459-x (PMC12297409; doi:10.1038/s41597-025-05459-x)
Supplement: Supplementary file 4 — Supplementary tables [file 41597_2025_5459_MOESM4_ESM.pdf]

Supplementary Tables

Supplementary Table 1	Summary of the ISA spectrophotometer operation, automatic cleaning, installation and manual cleaning
Supplementary Table 2	Naming convention used in the dataset
Supplementary Table 3	Range considered as valid for the data where the validity range check was performed.
Supplementary Table 4 (In supplementary File 1)	Organic chemicals analyzed during rain events.
Supplementary Table 5 (In supplementary File 1)	HPLC gradient used for chromatographic separation of the target analytes on an Acquity UPLC HSS T3 column.
Supplementary Table 6 (In supplementary File 1)	Settings for an Agilent 6495C triple quadrupole mass spectrometer.
Supplementary Table 7 (In supplementary File 1)	Acquisition method on an Agilent 6495C triple quadrupole mass spectrometer.
Supplementary Table 8 (In supplementary File 1)	List with all analyzed organic chemicals, their InChiKey for identification, the observed level of quantification (LOQ) and used isotope labelled standard used for quantification.
Supplementary Table 9 (In supplementary File 1)	List with relative recoveries of all spiked samples.
Supplementary Table 10 (In supplementary File 1)	List with matrix factors.
Supplementary Table 11 (In supplementary File 1)	List with limits of detection corrected with the respective matrix factors.
Supplementary Table 12 (In supplementary File 1)	List with measured concentrations and relative recoveries of the Pharma-Mix17.
Supplementary Table 13 (In supplementary File 1)	Measured concentrations of target analytes in field blinds.

Supplementary Table 1: Summary of the ISA spectrophotometer operation, automatic cleaning, installation and manual cleaning.

**Operation**	Measurement every 2 minutes with a pressurized air cleaning every 5 measurements
**Pressurized air cleaning**	10 s, with a waiting time of 10 s before the next measurement
**Sensor installation**	Diagonally at the bottom of the flume<br>Wooden aerodynamic element at the front<br>Packaging of the sensor cables in a cylindrical housing
**Sensor cleaning**	One to twice a week<br>Water jet, scrub and ethanol

Supplementary Table 2: Naming convention used in the dataset.

Sensor location	Sensor name	measured variables	unit
Catchment	pluvio2s	precipitation	mm
	kido	occupancy	-
Flume	isa	absorbance	_m
	isemax	ph	-
		nh4	v
		temperature	degc
	scan	absorbance	_m
		temperature	degc
	turbimax	turbidits	FNU
	mvx	Raw datacubes	-
		reflectance	-
Hall	cs2	flow	l_s
	e53	ec	us_m
	i3	level	mm
lab	lab	doc	mg_l
		po4	mg_l
		so4	mg_l
		nh4	mg_l
		nsol	mg_l
		toc	mg_l
		nsol	mg_l
		tss	mg_l
		turbidity	ntu
		organic chemicals	ng_l

Supplementary Table 3: Range considered as valid for the data where the validity range check was performed.

Sensor	Data name	Range considered as valid

MV.X	VNIR reflectance spectra	0.5-0.25 (at 600nm)
Spectrolyser	UV-vis absorbance spectra	10-1000 m^{-1} (at 255nm)
ISA	UV-vis absorbance spectra	0.02-1 (at 254nm)
Turbimax	Turbidity	20-1000 NTU
Nivus CS2	Sewer flow	20-900 L/s
Nivus i3	Sewer level	100-1000 mm

18
